# Supplementary material for: In situ captured antibacterial action of membrane-incising peptide lamellae
Source: Nat Commun. 2024 Apr 23;15:3424. doi: 10.1038/s41467-024-47708-4 (PMC11039730; doi:10.1038/s41467-024-47708-4)
Supplement: Supplementary file 1 — Supplementary Information [file 41467_2024_47708_MOESM1_ESM.pdf]

In Situ **Captured Antibacterial Action of Membrane-Incising Peptide Lamellae**

Kamal el Battioui,[1,5] Sohini Chakraborty,[1] András Wacha,[1] Dániel Molnár,[2,6] Mayra Quemé-Peña,[1,5] Imola Cs. Szigyártó,[1] Csenge Lilla Szabó,[4,5] Andrea Bodor,[4] Kata Horváti,[7] Gergő Gyulai,[7,8] Szilvia Bősze,[9] Judith Mihály,[1] Bálint Jezsó,[1] Loránd Romángszki,[1] Judit Tóth,[2,3] Zoltán Varga,[1] István Mándity,[1,10] Tünde Juhász,[1] Tamás Beke-Somfai[1,11]✉

[1] Institute of Materials and Environmental Chemistry, HUN-REN Research Centre for Natural Sciences, Budapest H-1117, Hungary
[2] Institute of Molecular Life Sciences, HUN-REN Research Centre for Natural Sciences, Budapest H-1117, Hungary
[3] Department of Applied Biotechnology and Food Sciences, Budapest University of Technology and Economics, Budapest H-1111, Hungary
[4] ELTE Eötvös Loránd University, Institute of Chemistry, Analytical and BioNMR Laboratory, Budapest H-1117, Hungary
[5] Hevesy György Ph.D. School of Chemistry, Eötvös Loránd University, Budapest H-1117, Hungary
[6] Doctoral School of Biology and Institute of Biology, Eötvös Loránd University, Budapest H-1117, Hungary
[7] MTA-HUN-REN TTK "Momentum" Peptide-Based Vaccines Research Group, Institute of Materials and Environmental Chemistry, Research Centre for Natural Sciences, Budapest H-1117, Hungary
[8] ELTE Eötvös Loránd University, Institute of Chemistry, Laboratory of Interfaces and Nanostructures, Budapest H-1117, Hungary
[9] HUN-REN ELTE Research Group of Peptide Chemistry, Hungarian Research Network, Eötvös Loránd University, Budapest, Hungary
[10] Department of Organic Chemistry, Faculty of Pharmacy, Semmelweis University, Budapest H-1092, Hungary
[11] Department of Chemistry and Chemical Engineering, Physical Chemistry, Chalmers University of Technology, Gothenburg SE-41296, Sweden

✉corresponding author: Tamás Beke-Somfai (e-mail address: beke-somfai.tamas@ttk.hu)

This PDF file includes:

Supplementary Figures 1 to 32
Supplementary Tables 1 to 7

Table of Contents:

1. **Extended Methods for MD Simulations.**
2. **Extended details on MD simulation results.**
3. **Lysine-Lysine distances obtained from MD simulations for monomeric and octameric 3K in the presence and absence of phosphate ions.**
4. **CD spectral features of 2K and 3K in different media support phosphate-assisted assembly formation.**
5. **Concentration dependence of 3K.**
6. **Titration of 3K in water with 0-10 mM PBS.**
7. **ATR-IR spectra of 2K and 3K in different media report on peptide conformational arrangement in the peptide-phosphate coassembly.**
8. **NMR detects disordered and monomeric 2K and 3K in pure water.**
9. **ANS binding capacity is higher for 3K and in phosphate media.**
10. **MD simulations assessing helix-forming affinity of 3K in the presence of phosphates.**
11. **Testing the effect of oligomerization on helix forming affinity for 3K by MD simulations.**
12. **Torsional angle distribution of β-amino acids throughout the 24-mer 3K simulation in phosphates.**
13. **Interaction of 2K and 3K with Suramin.**
14. **NS-TEM and cryo-EM images of 2K in PBS.**
15. **NS-TEM images of 3K in PBS.**
16. **Electron micrographs of 2K and 3K show solvent-dependent morphology.**
17. **Calculated spatial dimensions of 3K coassemblies.**

18. **Cryo-EM images of 3K validate the striped lamella morphology in solution.**
19. **Periodic repeat distance of the 3K-phosphate coassembly determined from SAXS scattering pattern.**
20. **AFM images of 3K-phosphate coassemblies reveal concentration dependent morphology variants.**
21. **liquid AFM images of 3K-PBS.**
22. **Schematic representation of bacterial lipopolysaccharides.**
23. **NS-TEM images of LPS treated with 3K.**
24. **ATR-IR spectra report on insertion of aligned peptides between the LPS chains in the 3K-LPS coassembly.**
25. **CD spectral pattern suggests minor peptide conformational changes upon binding to model vesicles.**
26. **CD spectral pattern of 3K in the presence of extracellular vesicles.**
27. **Antibacterial activity of 2K and 3K in the presence and absence of phosphate ions.**
28. **Electron micrographs of 3K treated *E. coli* cells show damaged cells and leakage.**
29. **Cryo-EM of 3K-treated *E. coli* cells.**
30. **Leakage Assays of 3K treated *E. coli* cells.**
31. **Cytotoxic activity of 3K in presence and absence of phosphate ions.**
32. **The salt-bridge network within the 3K-phosphate coassembly was assessed from MD simulation**.
33. **RP-HPLC chromatograms and HRMS spectra of purified 2K and 3K.**

Extended Methods for MD Simulations:

Molecular dynamics (MD) simulations have been carried out using version 2021.6 of the GROMACS software,[1,2] with the CHARMM force field extended for β-peptides.[3] The molecular models of our foldamers were prepared in the required conformation using PyMOL version 2.4, using the pmlbeta extension.[4] After a short energy minimization with the steepest descent algorithm to resolve residual strains in the molecule, the peptides were put in a cubic box of CHARMM-modified TIP3P water. Due to the absence of the required parameters for hydrogenphosphate in the CHARMM force field, divalent methylphosphate (residue topology MP_2) was used instead. In addition to the minimum number of sodium and/or chloride ions required to neutralize the system, more NaCl was added in a concentration equivalent to 150 mM. Next, a short steepest descent energy minimization was executed on the solvent (water and ions; the heavy atoms of the peptides being kept in fixed positions by applying harmonic restraining potentials of 1000 kJ/nm), in order to ensure good solvation of the macromolecule and to get rid of voids and steric clashes between atoms. The temperature of the system was set to 300 K in a 100 ps simulation run in the NVT ensemble using a Berendsen thermostat with 0.1 ps coupling constant.[5] Equilibration in the NpT ensemble followed using an isotropic Berendsen barostat set at 1 bar, with 2 ps coupling time. Because the Berendsen thermostat and barostat do not sample the correct statistical ensemble, during the production run the velocity-rescaling thermostat with stochastic extension[6] and the Parrinello-Rahman barostat[7] was used. Long-range electrostatics was accounted for by the particle mesh Ewald algorithm implemented in the GROMACS program, the short-range Coulomb cut-off being 1.2 nm, dictated by the CHARMM force field. Based on the extended in depth testing analysis related to our developed extension to the CHARMM force field for describing β-peptide folding, unfolding, and also β-peptide lamellin formation[3,8,9], it was identified that 500 ns simulation time is sufficiently long to allow adequate sampling of the conformational space of the molecules, especially with respect to the side chain orientations of our studied systems (Supplementary Fig.1). Accordingly, after preliminary test simulations, one MD production run of 500 ns simulation time were carried out to provide qualitative insight on changes of the lysine side chain dynamics once phosphates are also present in the system (Supplementary Fig.1). Based on the results of the above simulations where the formation of spontaneous association was studied, as well as on our surmises from transmission electron micrographs, a larger associate of 24 3K monomers was assembled and an MD simulation was performed with the similar setup as above.
In addition to the above, six more simulations were performed to rule out helical conformations of the 3K peptide. In each of these runs, a single peptide strand was prepared in a certain helical conformation ($H10_M$, $H10_P$, $H12_M$, $H12_P$, $H14_M$ and $H14_P$). To avoid early unfolding of the peptides due to steric clashes, harmonic distance restraint potentials (2000 kJ/mol strength, equilibrium distance 0.22 nm) were placed between all hydrogen and oxygen atom pairs responsible for stabilizing the given helix. After the same minimization and equilibration procedure as detailed above, production runs were performed for 500 ns each, with the distance restraints in place. At this point, all restraints were lifted and the simulations were continued for another 500 ns. All the simulation files can be viewed at: https://doi.org/10.5281/zenodo.8363528.

Extended details on MD simulation results:

To address whether helical conformation could be reached for these systems, we have performed MD simulations. The available secondary structures are somewhat limited for these short peptides with alternating chirality and repetitive sequences. However, the strong helix-forming affinity of β-peptides is well known, thus we tested on 3K whether phosphate ions could stabilize it in a helix conformation. We employed our developed force field for β-peptides that could accurately predict the correct fold for several sequences,[3,8,10] and performed six simulations on both left- and right handed helices with forward and also backward H-bond patterns. Under fully relaxed conditions, all these helices quickly unfolded into the extended zig-zag conformations (Supplementary Fig.9). Further on, to test whether helix formation could be stabilized by oligomeric assemblies with phosphates, a new 1 µs MD simulation was performed with eight $H10_P$ helices in the presence of phosphates and holding the helical conformation for 500 ns to allow the system for helical oligomerization (Supplementary Fig.10). Upon releasing these restraints, the individual helices again unfolded into extended zig-zag conformations that started to form intermolecular H-bonds with each other. These all strongly indicate that the inherent structural features of 3K make helical conformations unfavorable. To address formation of larger sheet-like assemblies, the initial MD simulations on octameric lamellin-3K models (Supplementary Fig.1b), were used to build tetracosameric (24-mer) models for additional unrestrained-MD simulations (Fig.1c,e). These MD runs clearly displayed double arrays of lamellin-3K in parallel orientation, where intermolecular H-bonds stabilized the assembly along the long axis while the two arrays formed a hydrophobic core composed of the leucine side chains. The double arrays of lamellin-3K are connected by a narrow layer of phosphate ions that coordinate lysine side chains. The

accumulation of phosphate ions on the outer edges of the assembled peptide arrays suggests that in principle this oligomeric motif could be repeated in the coassembly. The individual 3K conformations demonstrate a zig-zag type sheet formation, that is similar to the theoretical predictions reported by Seebach et al.[11] and by us.[12,13] However, interestingly, we only see a parallel orientation for lamellin-3K with alternating torsional angles in the neighbouring hydrogen bonded amino acids along the long axis of the sheet (*i.e.* j:-120, q: 60, y: -120 and j: 120, q: -60, y: 120) (Supplementary Fig.11) This conformation lies closest to the parallel sheet orientation suggested by Martinek et al.[14], which was composed by cyclic ACPC residues with alternating chirality.

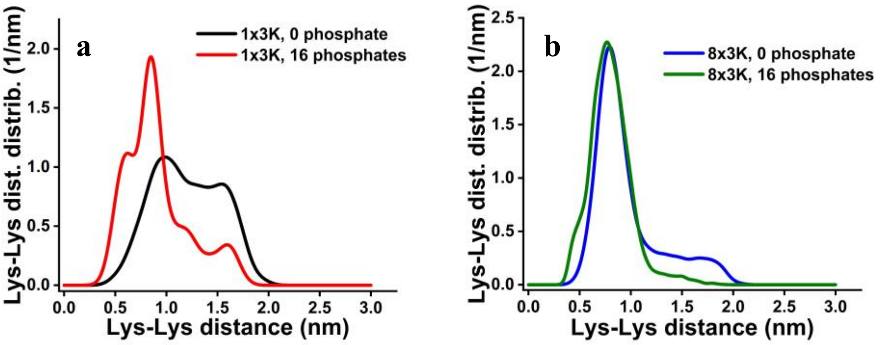

Supplementary Figure 1: Lysine-Lysine distances obtained from MD simulations for monomeric and octameric 3K in the presence and absence of phosphate ions. Lysine-Lysine distances were calculated for simulations with monomeric (a) or octameric (b) 3K with and without 16 divalent phosphate ions. Lys3-Lys5 and Lys5-Lys7 distances were averaged for the entire simulation time to provide a qualitative insight on distance distribution. For more details see the extended sections on MD methods and simulations in the Supporting Information.

Supplementary Table 1: Summary of the molecular dynamic simulations carried out in the present work.

# Peptide	# $CH_3PO_4^{2-}$	# H_2O	# Na^+/Cl^-	Initial box size	Initial peptide conformation	Total # of atoms
1× 2K-lamellin	0	3983	11/13	$5 \times 5 \times 5$ nm^3	zig-zag	12126
1× 2K-lamellin	16	3876	41/11	$5 \times 5 \times 5$ nm^3	zig-zag	11977
1× 3K-lamellin	0	3962	11/14	$5 \times 5 \times 5$ nm^3	zig-zag	12111
1× 3K-lamellin	16	3863	40/11	$5 \times 5 \times 5$ nm^3	zig-zag	11984
8× 3K-lamellin	0	10601	31/55	$7 \times 7 \times 7$ nm^3	Zig-zag, random relative position and orentation	33489
8× 3K-lamellin	16	10568	39/31	$7 \times 7 \times 7$ nm^3	Zig-zag, random relative position and orentation	33518
24× 3K-lamellin	100	5816	128/0	$5.9 \times 7.0 \times 5.2$ nm^3	4 copies of the 6-mer formed from zig-zag conformations in the 8× 3K-lamellin simulation with 16 phosphates	23276
1×3K-lamellin	50	3647	108/11	4.8 nm	$H10_M$	11710
1×3K-lamellin	50	3647	108/11	4.8 nm	$H10_P$	11710
1×3K-lamellin	50	3632	108/11	4.8 nm	$H12_M$	11665
1×3K-lamellin	50	3649	108/11	4.8 nm	$H12_P$	11716
1×3K-lamellin	50	3657	108/11	4.8 nm	$H14_M$	11740
1×3K-lamellin	50	3628	108/11	4.8 nm	$H14_P$	11653

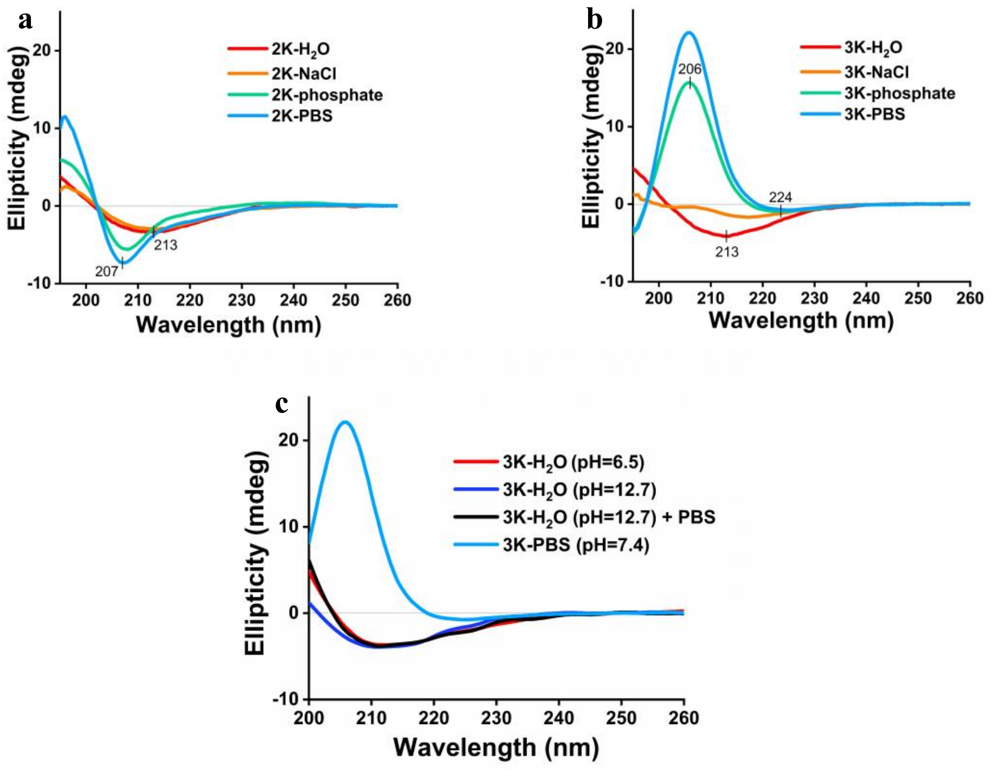

Supplementary Figure 2: CD spectral features of 2K and 3K in different media support phosphate-assisted assembly formation. Circular dichroism spectra were taken at 125 μM peptide concentration in different media (H_2O, pH 6.5; NaCl 150 mM, pH 6.5; 10 mM phosphate, pH 7.4; and PBS 10 mM, pH 7.4) at 25 °C for 2K (a), and 3K (b). For comparison, spectra of 3K were also recorded in water (pH=6.5) and the pH was adjusted to 12.7 using 10 M NaOH followed by the addition of 0.5 mM PBS at pH 12.7 (c). The figure shows representative CD spectra of at least three independent measurements. The general CD signature obtained for 2K in water and NaCl showed a broad negative peak with a minimum at ~ 213 nm, and a maximum at ~ 190 nm, which points to the predominance of a random coil structure, based on previous results with similar sequences.[9] In contrast, in phosphate solutions 2K underwent a conformational change towards a more ordered structure, as indicated by an extra, sharp negative peak component developing at ~ 207 nm, which became more marked in PBS. For 3K in phosphate-free conditions, a disordered conformation similar to that of 2K can be assigned, however, the appearance of an intense maximum at 206 nm in phosphate-containing solutions, particularly in PBS, points to formation of highly ordered assemblies.

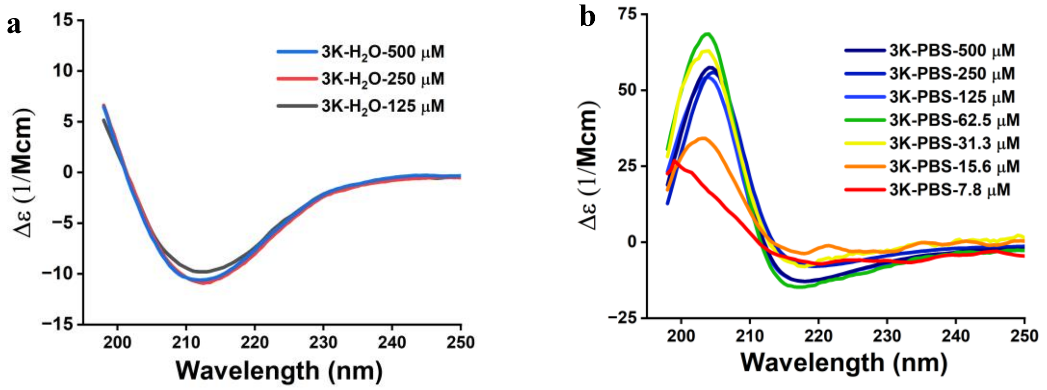

Supplementary Figure 3: Concentration dependence of 3K. Concentration-dependent circular dichroism spectra of 3K in (a) H_2O, pH 6.5 at 25 °C, (b) PBS 10 mM, pH 7.4. In water at 500, 250 and 125 μM concentrations, 3K show similar CD signature detected with a minimum at ~ 212 nm, which suggest the presence of the random coil structure. In contrast, in PBS (b), the formation of ordered assemblies can be detected with the gradual appearance of the maximum at ~ 205 nm.

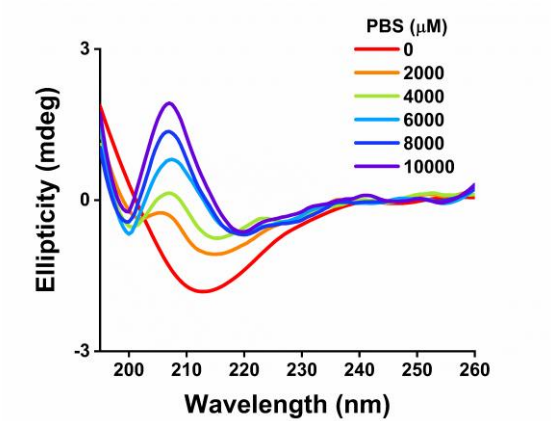

Supplementary Figure 4: Titration of 3K in water with 0-10 mM PBS. Additionally, to follow the formation of ordered assemblies in PBS, 3K (125 μM) in water was titrated with increased concentration of 2-10 mM PBS. Here, the transition from random coil structure at 0 μM of PBS towards more ordered assemblies can be trailed effectively.

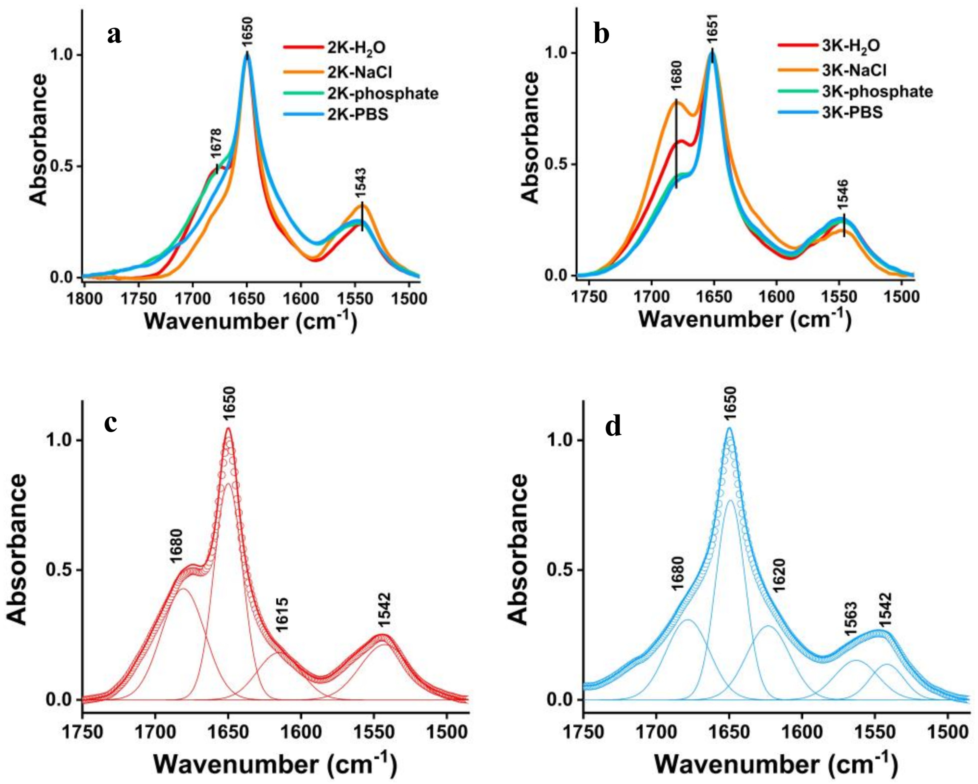

Supplementary Figure 5: ATR-IR spectra of 2K and 3K in different media report on peptide conformational arrangement in the peptide-phosphate coassembly. ATR-IR spectra of (a) 2K and (b) 3K were recorded for dry film samples obtained from solutions as described in Supplementary Fig.2. The IR spectra of 2K and 3K exhibit peculiar bands, namely amide I at 1600 - 1700 cm^{-1} and amide II at 1500 - 1600 cm^{-1}, characteristic for backbone amide bonds in peptides and proteins. For both peptides, the amide I band could be deconvoluted to three main components (see the representative examples for (c) 2K-water and (d)2K-PBS). Based on band width, and the similarity to IR pattern of strand forming β-peptides and a peptide with close sequence build-up to 2K (peptide 5 in Szigyarto et al.[9]), the strongest amide I component centered at 1650 cm^{-1} could likely be assigned to an extended conformation with intramolecular H-bonds. Likewise, the shoulder at ~ 1680 cm^{-1} can be assigned to turn motives[15] or backbone amide C=O groups not involved as acceptors in H-bonding.[14,16] The low wavenumber band component at 1615-1620 cm^{-1} is indicative of intermolecular H-bonding. For 2K, the higher relative intensity of the shoulder at ~ 1680 cm^{-1} suggests a looser structure in non-phosphate media while the intensity gain of the component at 1615-1620 cm^{-1} is indicative of higher association of the peptide chains in phosphate buffers. For 3K, the non-H-bonded amide C=O population is significantly reduced in the presence of phosphate ions, which is in line with more H-bonds formed in the phosphate-assisted coassemblies.

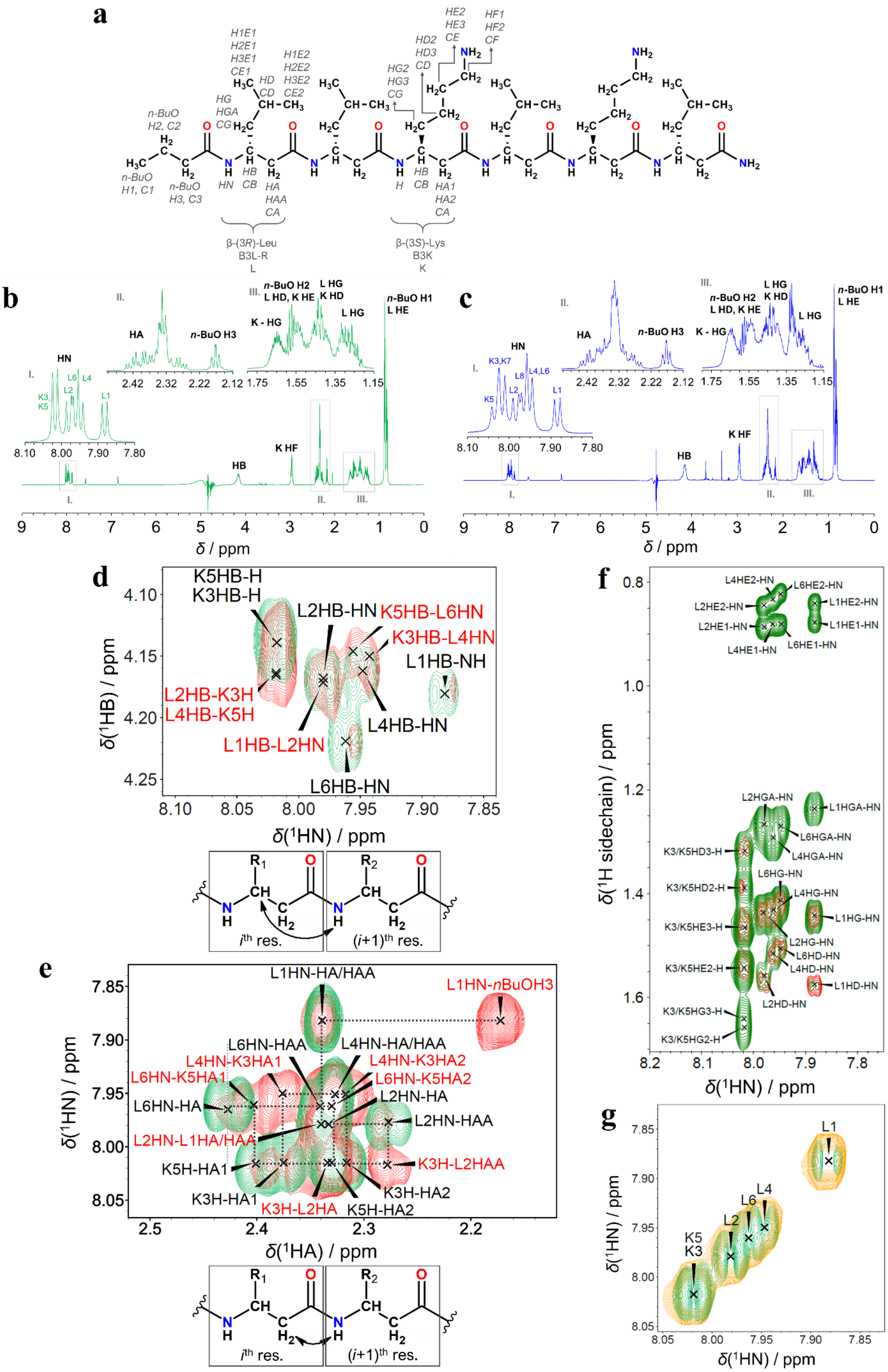
a
n-BuO H2, C2
n-BuO H1, C1
n-BuO H3, C3
HN
HB CB
HA HAA CA
HG HGA CG
HD CD
H1E1 H2E1 H3E1 CE1
H1E2 H2E2 H3E2 CE2
HG2 HG3 CG
HD2 HD3 CD
HE2 HE3 CE
HF1 HF2 CF
H
HB CB
HA1 HA2 CA
β-(3R)-Leu
B3L-R
L
β-(3S)-Lys
B3K
K
b
HN
K3, K5
L6 L4
L2
L1
HA
n-BuO H3
n-BuO H2
L HD, K HE
L HG
K HD
K - HG
L HG
n-BuO H1
L HE
HB
K HF
I.
II.
III.
δ / ppm
c
HN
K3,K7
K5
L8
L2
L4,L6
L1
HA
n-BuO H3
n-BuO H2
L HD, K HE
L HG
K HD
K - HG
L HG
n-BuO H1
L HE
HB
K HF
I.
II.
III.
δ / ppm
d
K5HB-H
K3HB-H
L2HB-HN
K5HB-L6HN
K3HB-L4HN
L1HB-NH
L2HB-K3H
L4HB-K5H
L1HB-L2HN
L4HB-HN
L6HB-HN
δ(¹HB) / ppm
δ(¹HN) / ppm
ith res.
(i+1)th res.
e
L1HN-HA/HAA
L1HN-nBuOH3
L6HN-HAA
L4HN-HA/HAA
L4HN-K3HA1
L4HN-K3HA2
L6HN-K5HA1
L6HN-K5HA2
L6HN-HA
L2HN-HA
L2HN-HAA
L2HN-L1HA/HAA
K5H-HA1
K3H-L2HAA
K3H-HA1
K3H-HA2
K3H-L2HA
K5H-HA2
δ(¹HN) / ppm
δ(¹HA) / ppm
ith res.
(i+1)th res.
f
L4HE2-HN
L6HE2-HN
L2HE2-HN
L1HE2-HN
L2HE1-HN
L1HE1-HN
L4HE1-HN
L6HE1-HN
L2HGA-HN
L1HGA-HN
L6HGA-HN
K3/K5HD3-H
L4HGA-HN
L6HG-HN
K3/K5HD2-H
L4HG-HN
K3/K5HE3-H
L1HG-HN
L2HG-HN
L6HD-HN
K3/K5HE2-H
L4HD-HN
L1HD-HN
L2HD-HN
K3/K5HG3-H
K3/K5HG2-H
δ(¹H sidechain) / ppm
δ(¹HN) / ppm
g
L1
L4
L6
L2
K5
K3
δ(¹HN) / ppm
δ(¹HN) / ppm

Supplementary Figure 6: NMR detects disordered and monomeric 2K and 3K in pure water. a) The nomenclature of ^{1}H chemical environments used in this study is shown on the example of 2K. b) and c) show 1D ^{1}H spectrum at 298 K of 2K (in green) and 3K (in blue), respectively. I, II and III regions are magnified, and the assignment of peak groups is indicated. d-g) Regions of overlaid ^{1}H-^{1}H TOCSY (green) and ^{1}H-^{1}H ROESY (red) for 2K, indicating sequential connectivities (d, e), and the lack of long-range (f) and H^{N}-H^{N} (g) crosspeaks. NMR chemical shifts in water were deposited to BMRB with entry numbers 51854 (for 2K) and 51856 (for 3K).

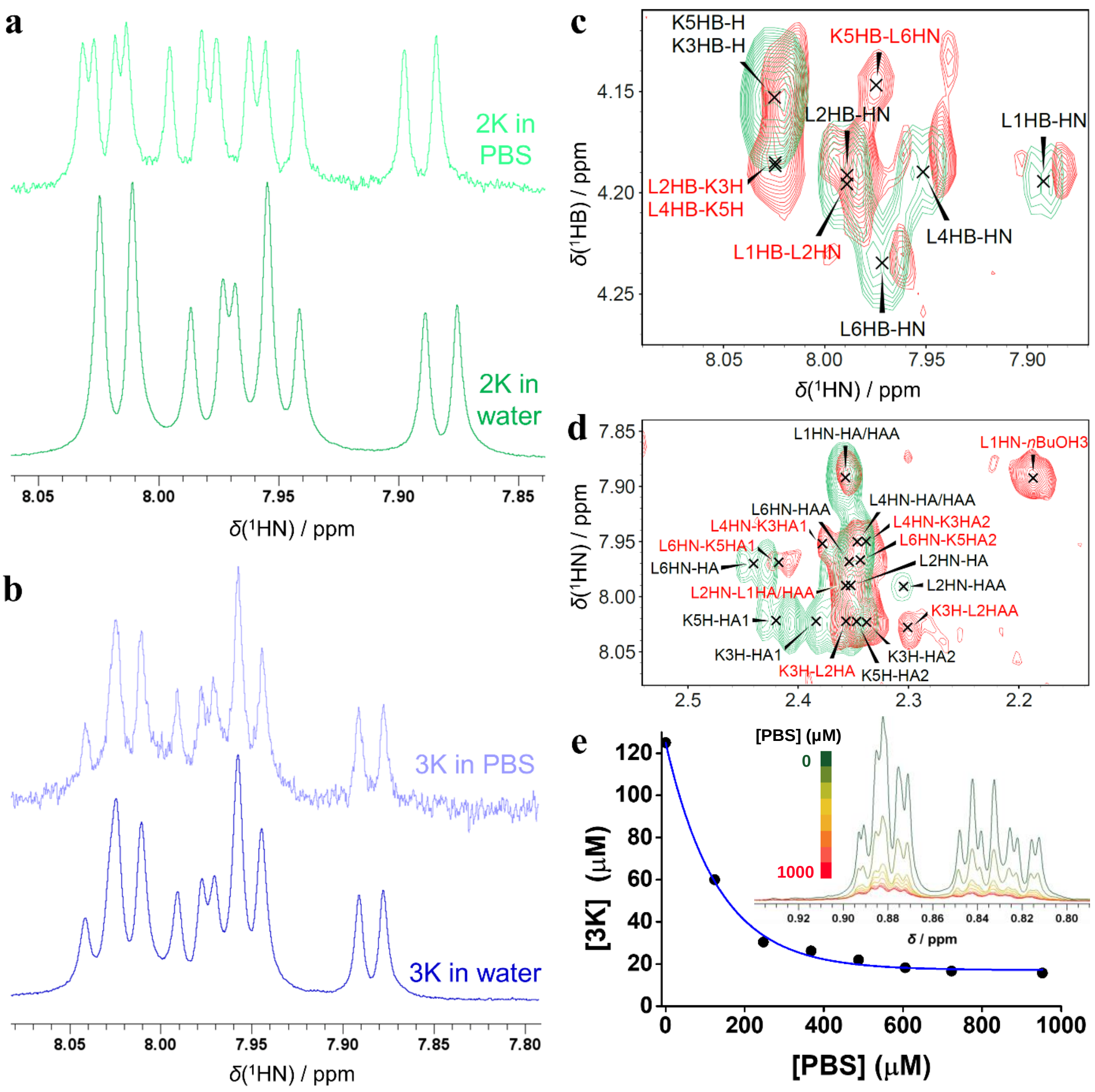

Supplementary Figure 7: a) and b) Overlay of 1D ^{1}H spectra in water (c = 1 mM, dark colour) and in PBS (light colour) for 2K (green) and 3K (blue), respectively. Concentration in PBS was ~ 250 μM for 2K and ~ 20 μM for 3K. c) and d) Regions of overlaid ^{1}H-^{1}H TOCSY (green) and ^{1}H-^{1}H ROESY (red) for 2K, indicating sequential connectivities. e) Variation of the 3K concentration during PBS titration determined from the integration of leucine methyl signal. Starting concentration of 3K in pure water was 125 μM (inset shows the 1D ^{1}H NMR spectra of 3K (125 μM) upon increasing PBS concentration).

Supplementary Table 2: PFG-NMR experiment parameters, and calculated diffusion coefficients of 2K and 3K under various conditions.

Diffusion coefficients were determined according to the Stejskal-Tanner equation,

$$I = I_0 \exp\left(-D\gamma^2 G^2 \delta^2 (\Delta - \delta/3)\right)$$

where I is the signal integral, I_0 is the integral without using gradient pulses, D is the diffusion coefficient, γ is the magnetogyric ratio of ^{1}H, G is the gradient strength, δ is the gradient pulse length, Δ is the diffusion delay. Diffusion coefficients determined with PFG-NMR supported the presence of disordered monomers in the solution, both in water and in PBS. Data are represented as mean ± SD (n=3 independent experiments).

Peptide	c / mM	Medium	δ / ms	Δ / ms	D / m^2s^{-1}
2K	1.0	H_2O, 10% D_2O, pH = 6.7	2.0	150	$(2.94 \pm 0.03) \cdot 10^{-10}$
	0.25	PBS, 10% D_2O, pH = 7.2	2.2	120	$(2.90 \pm 0.06) \cdot 10^{-10}$
3K	0.93	H_2O, 10% D_2O, pH = 7.1	2.2	150	$(2.59 \pm 0.05) \cdot 10^{-10}$
	0.125*	PBS 10 mM 6% D_2O, pH = 7.3	2.2	150	$(2.80 \pm 0.04) \cdot 10^{-10}$
	0.125	H_2O, 10% D_2O	-	-	-
	0.125*	PBS 0.125 mM, 10% D_2O	-	-	-
	0.125*	PBS 0.250 mM, 10% D_2O	2.2	150	$(2.66 \pm 0.06) \cdot 10^{-10}$
	0.125*	PBS 0.375 mM, 10% D_2O	-	-	-
	0.125*	PBS 0.500 mM, 10% D_2O	2.2	150	$(2.82 \pm 0.05) \cdot 10^{-10}$
	0.125*	PBS 0.625 mM, 10% D_2O	-	-	-
	0.125*	PBS 0.750 mM, 10% D_2O	-	-	-
	0.125*	PBS 1.00 mM, 10% D_2O	-	-	-

* coassembly formation detected

Supplementary Table 3: Chemical shifts of backbone H^N, H^α and H^β atoms, as well as $^3J_{HN\text{-}H\beta}$ and $^3J_{H\beta\text{-}H\alpha}$ scalar couplings.

2K	L1	L2	K3	L4	K5	L6
$\delta(H^N)$ / ppm	7.88	7.98	8.02	7.95	8.02	7.96
$\delta(H^\beta)$ / ppm	4.18	4.17	4.13	4.16	4.14	4.22
$\delta(H^{\alpha 1})$ / ppm	2.34	2.34	2.37*	2.33	2.39*	2.42*
$\delta(H^{\alpha 2})$ / ppm	2.34	2.28*	2.33	2.32	2.32	2.33
$\delta(H^{\alpha 1}) - \delta(H^{\alpha 2})$ / ppm	0.00	0.06	0.04	0.01	0.07	0.09
$^3J_{HN\text{-}H\beta}$ / Hz	9.3	9.5	9.4	9.3	9.4	9.3
$^3J_{H\beta\text{-}H\alpha i}$	N.A.	6.5	5.4	N.A.	5.7	6.0

3K	L1	L2	K3	L4	K5	L6	K7	L8
$\delta(H^N)$ / ppm	7.88	7.98	8.02	7.95	8.04	7.95	8.02	7.97
$\delta(H^\beta)$ / ppm	4.18	4.17	4.13	4.16	4.14	4.16	4.13	4.22
$\delta(H^{\alpha 1})$ / ppm	2.34	2.34	2.39	2.33	2.38	2.34	2.38	2.42*
$\delta(H^{\alpha 2})$ / ppm	2.34	2.27*	2.32	2.31	2.33	2.33	2.33	2.33
$\delta(H^{\alpha 1}) - \delta(H^{\alpha 2})$ / ppm	0.00	0.07	0.05	0.02	0.05	0.01	0.05	0.09
$^3J_{HN\text{-}H\beta}$ / Hz	9.3	9.4	9.7	9.3	9.5	9.3	9.7	9.6
$^3J_{H\beta\text{-}H\alpha i}$	N.A.	6.7	N.A.	N.A.	N.A.	N.A.	N.A.	6.1

* indicates the H^α for which the $^3J_{H\beta\text{-}H\alpha}$ coupling constant was determined

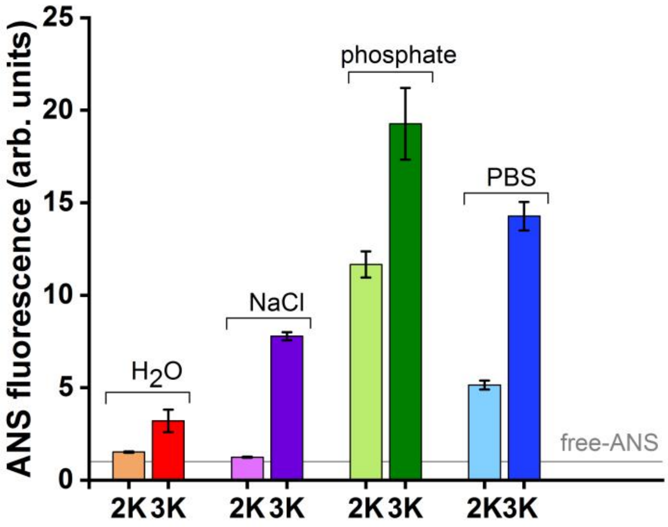

Supplementary Figure 8: ANS binding capacity is higher for 3K and in phosphate media. ANS binding of 2K and 3K (125 µM) were tested in the indicated media using the ANS probe at 2.5 µM. The emission intensities at the maxima were read, and values were normalized for that of unbound ANS. Gray line highlights the reference signal of free ANS. In all tested media, higher ANS binding was detected for 3K over 2K. Further on, higher ANS fluorescence was observed in the presence of phosphate ions compared to non-phosphate conditions. Error bars represent the standard deviation of the mean for three independent experiment sets.

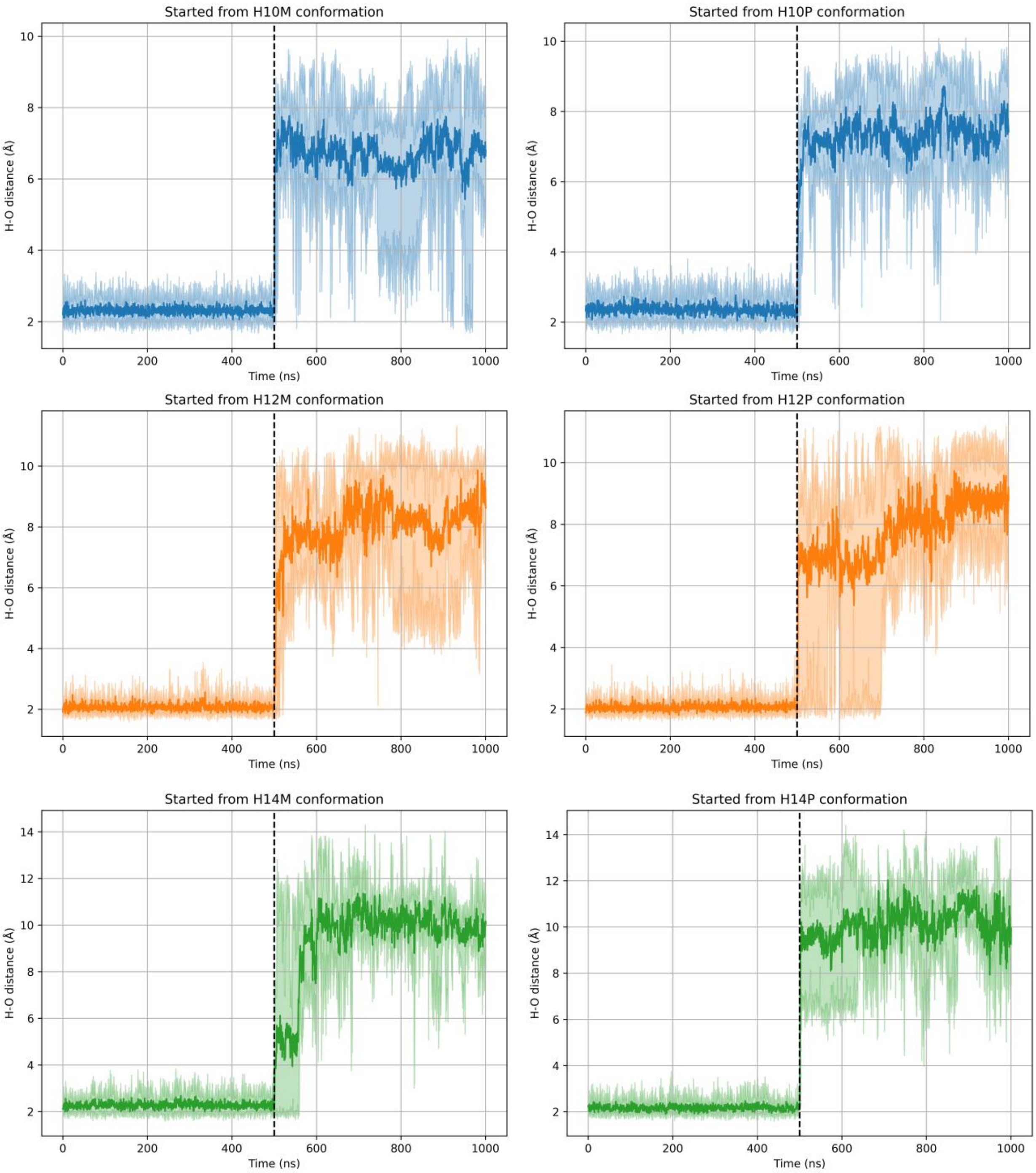

Supplementary Figure 9: MD simulations assessing helix-forming affinity of 3K in the presence of phosphates. Initial peptide hydrogen bond distances were set between the participating hydrogen and oxygen atoms representative for the particular type of helix tested. Restraints on these distances were applied to keep the secondary structure up to 500 ns of the simulation, allowing the phosphates to stabilize the fold. Restraints were released after 500 ns (dashed vertical line). Trajectories of all simulations were tested for formation of any other type of helical morphology, none of them showed refolding to another helical conformation. P and M represent, right- and left-handed helices, respectively. Data points are given for each nanosecond frame and the darker colour refers to the running average.

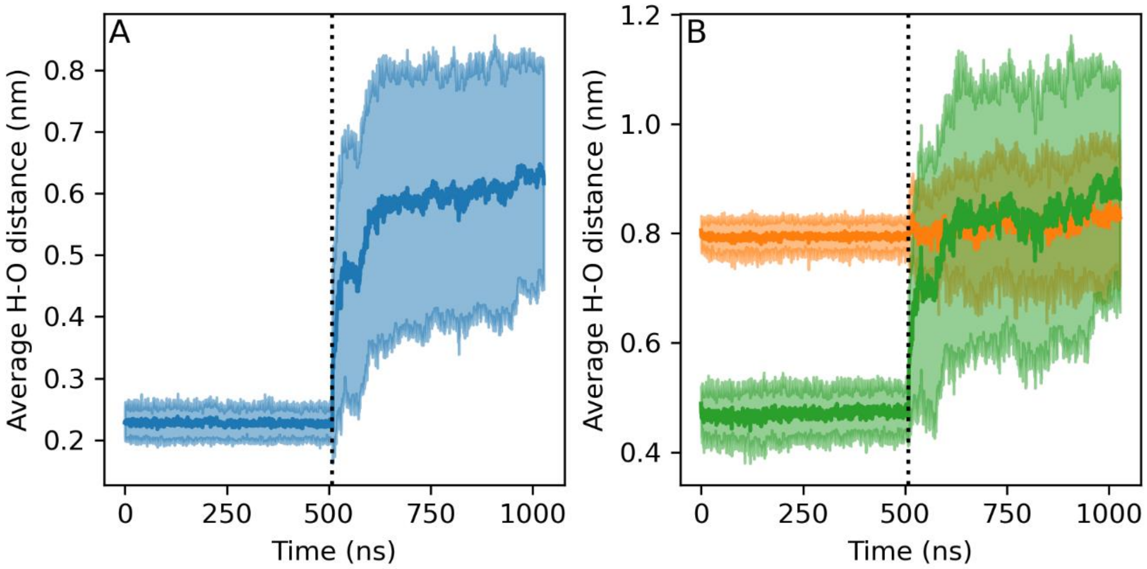

Supplementary Figure 10: Testing the effect of oligomerization on helix forming affinity for 3K by MD simulations. A: Average hydrogen bond distance (O and H bridge atoms) in eight 3K molecules folded into H10$_P$ helical conformation simulated in the presence of phosphates. Top and bottom bright blued values are the maxima and minima observed, respectively, while the dark blue line represents the average distances. Initial 500 ns contained distances restraints keeping all eight 3K molecules in H10 helical conformation. The restraints were removed after 500 ns (dotted vertical line). B: Bridge atom distances for H12 (orange) and H14 (green) were also calculated for the same simulation as described in A. These values indicate that after releasing the restraints the H10$_P$ helices did not fold into any alternative helical forms. Data points are given for each nanosecond frame and the darker colour refers to the running average.

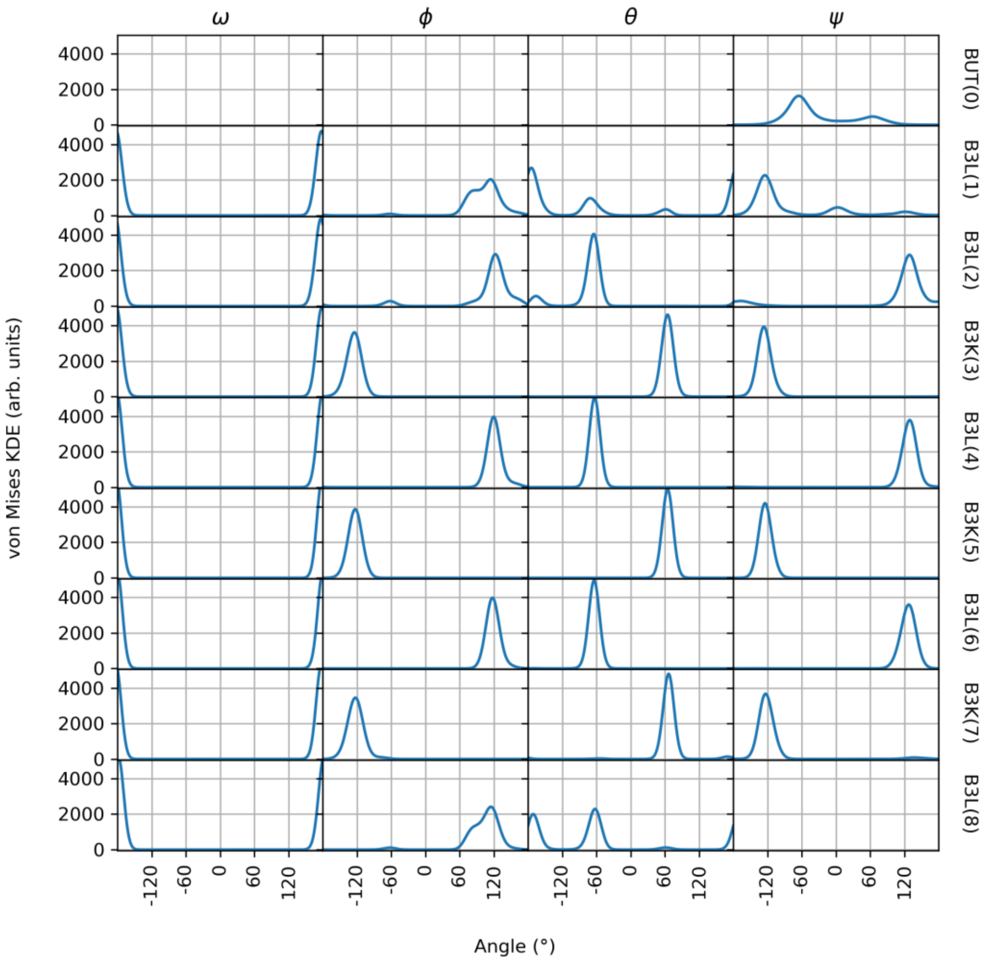

Supplementary Figure 11: Torsional angle distribution of β-amino acids throughout the 24-mer 3K simulation in phosphates. Values for each amino acid in the sequences are averaged over the 24 3K molecules. Angle distributions are derived from von Mises kerned density estimates (KDE). Residue numbering starts by 0, representing the N-terminal butyl protecting group and continues with B3L(1), B3L(2), etc., according to the sequences displayed on Fig.1a. For the central residues (2-7 in the sequence), an alternating pattern of −120, +60, −120 (residue i) and 120, −60, 120 (residue i+1), can be observed for the φ, θ and ψ torsional angles[17] of the peptide backbone, representing a zig-zag type secondary structure. ω displays the torsional angle of the trans peptide bonds.

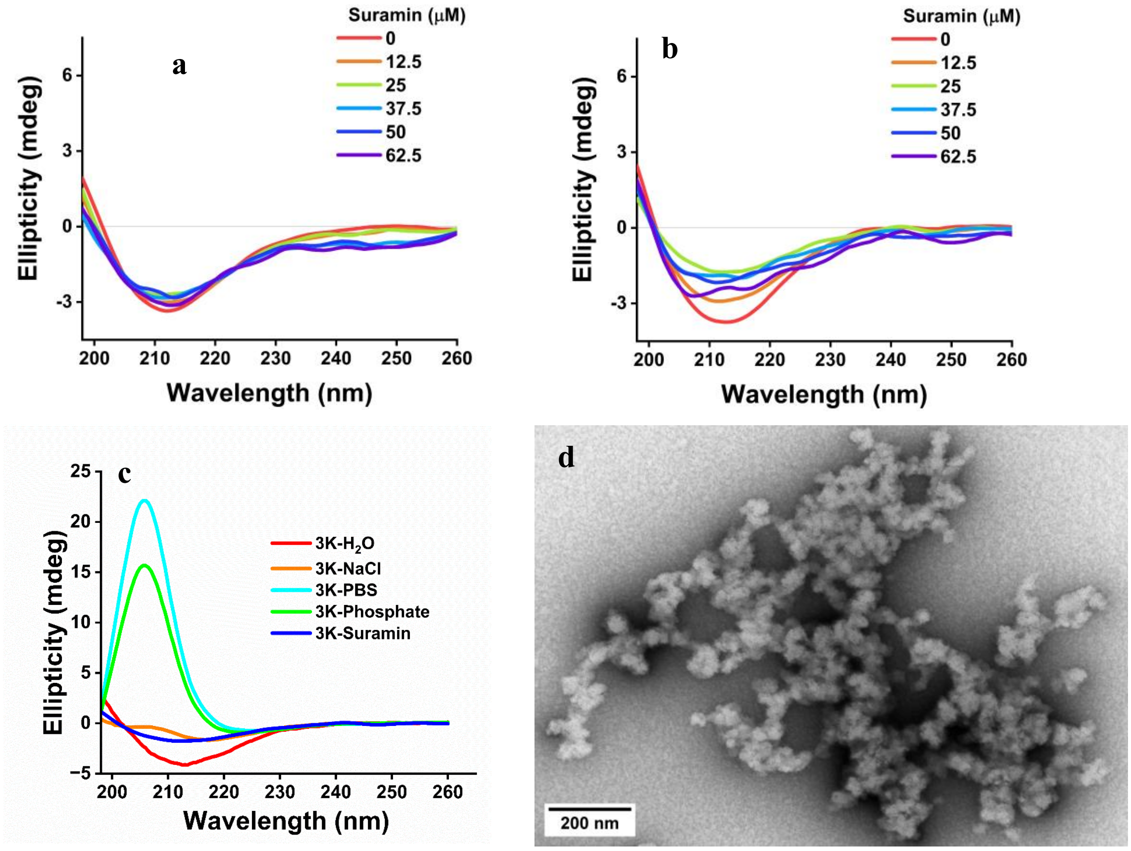

Supplementary Figure 12: Interaction of 2K and 3K with Suramin. CD spectra of (a) 2K and (b) 3K upon titration with suramin. (c) Comparison of CD spectra of 125 μM 3K in H_2O, pH 6.5; NaCl 150 mM, pH 6.5; 10 mM phosphate, pH 7.4; PBS 10 mM, pH 7.4 and suramin 25 μM at 25 °C. Spectra of both 2K and 3K display minima at ~ 213 nm and maxima at ~ 190 nm. The CD signature is similar to that obtained in water and NaCl suggesting the lack of an ordered structure observed in phosphate-containing solutions. (d) Negative stain-TEM of 3K-water complexed with suramin showing aggregates of sphere-like building blocks.

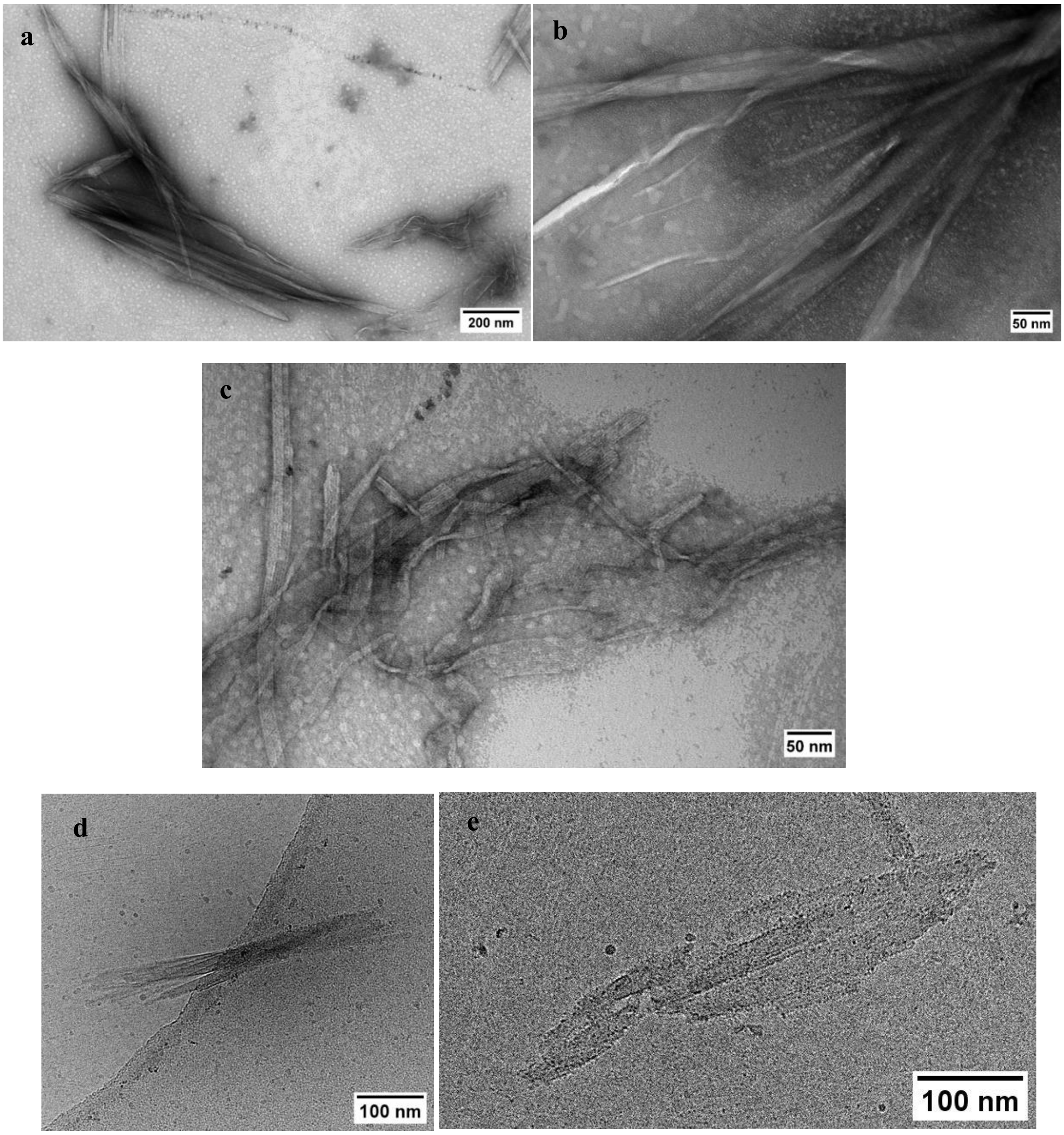

Supplementary Figure 13: EM images of (a-c) 2K (125 µM) in PBS stained with uranyl-acetate (NS-TEM) and cryo-EM images of 2K (125 µM) in PBS. 2K forms (up to micrometer) long, bundled, fibrillary structures. The presence of striped morphology is clearly observed in (c); however, these are longer, less ordered and more flexible than 3K in PBS. The morphologies of vitrified samples (d,e), obtained by cryo-EM resemble the characteristic striped fibrillar morphology observed in NS-TEM images.

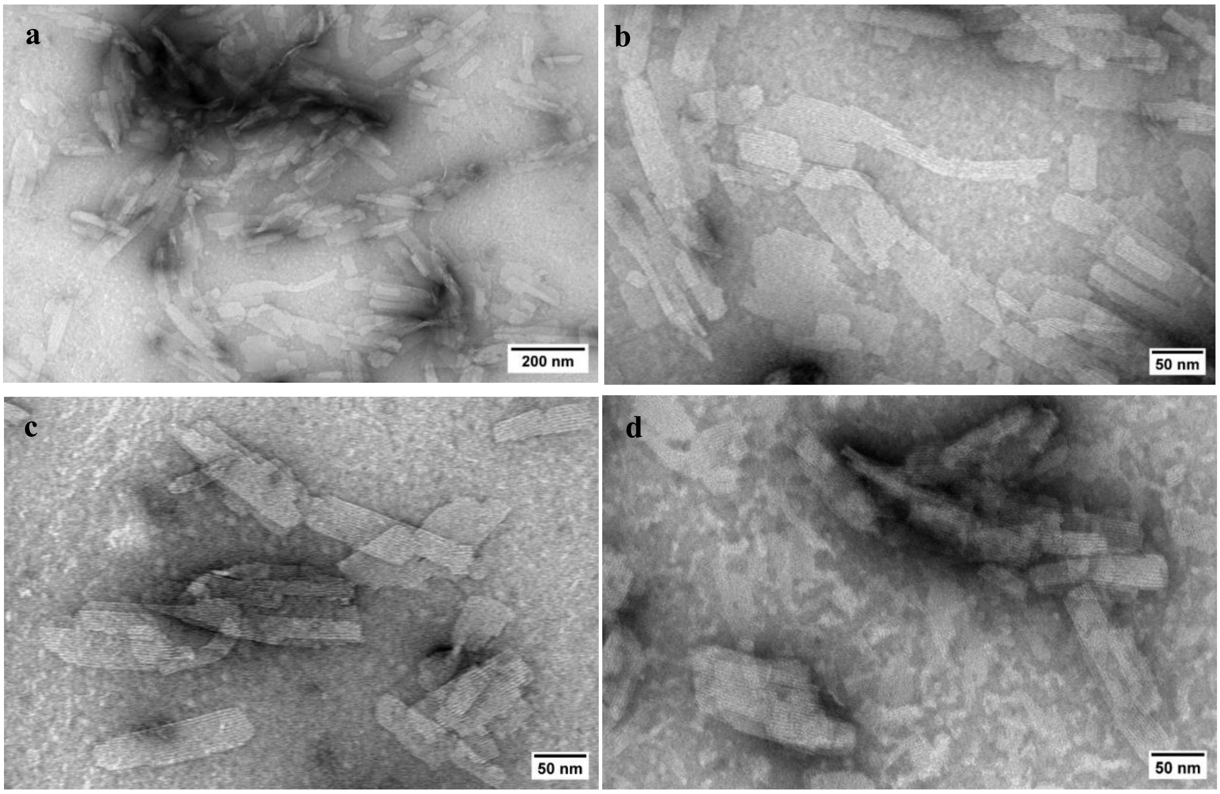

Supplementary Figure 14: NS-TEM images of (a-c) 3K (125 µM) in PBS stained with uranyl-acetate. 3K shows shorter, rather edged, sheet-like morphology, (d) NS-TEM images of 3K (125 µM) in PBS stained with phosphotungstic acid. The same kind of lamellar morphology can be obtained with both staining agents for 3K in PBS.

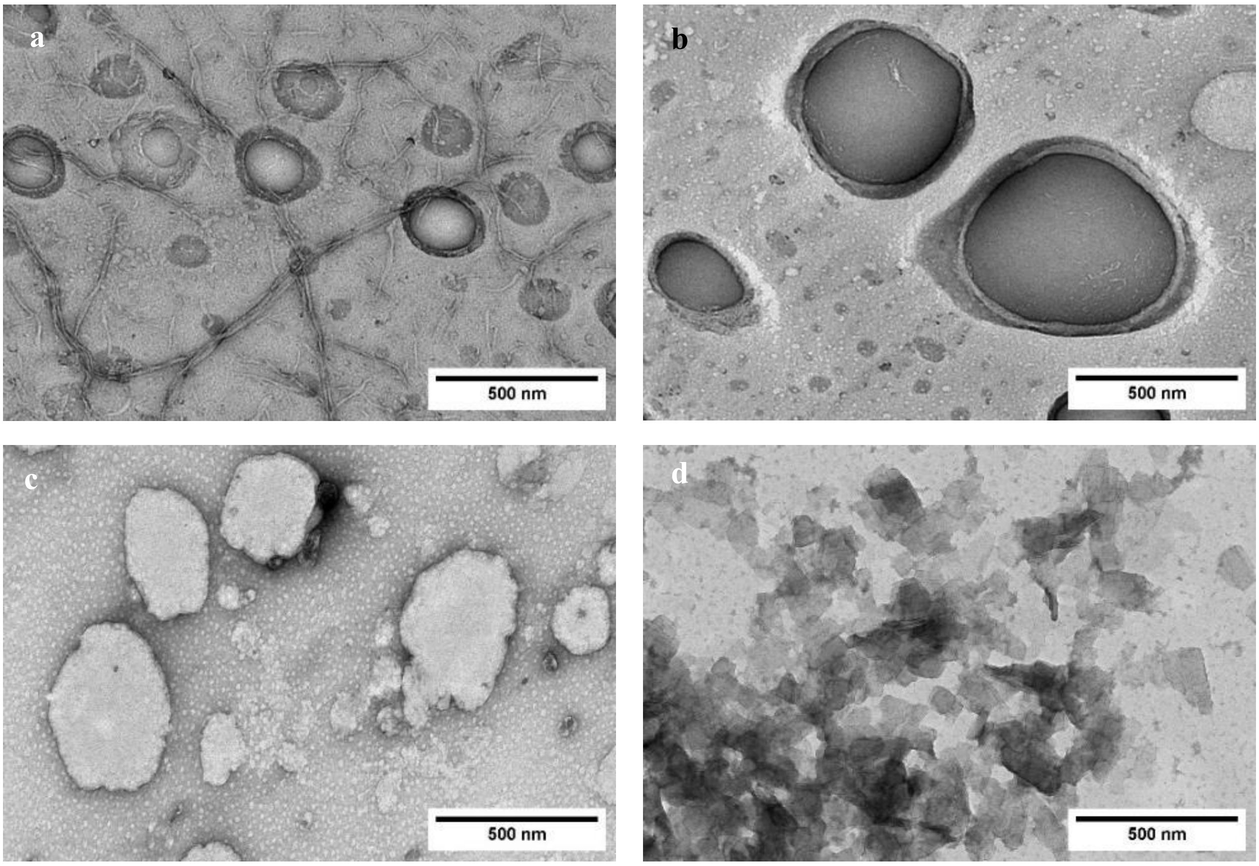

Supplementary Figure 15: NS-TEM images of (a) 2K and (b) 3K samples prepared in NaCl solution. NS-TEM images of (c) 2K and (d) 3K samples prepared at 125 μM in phosphate solution.

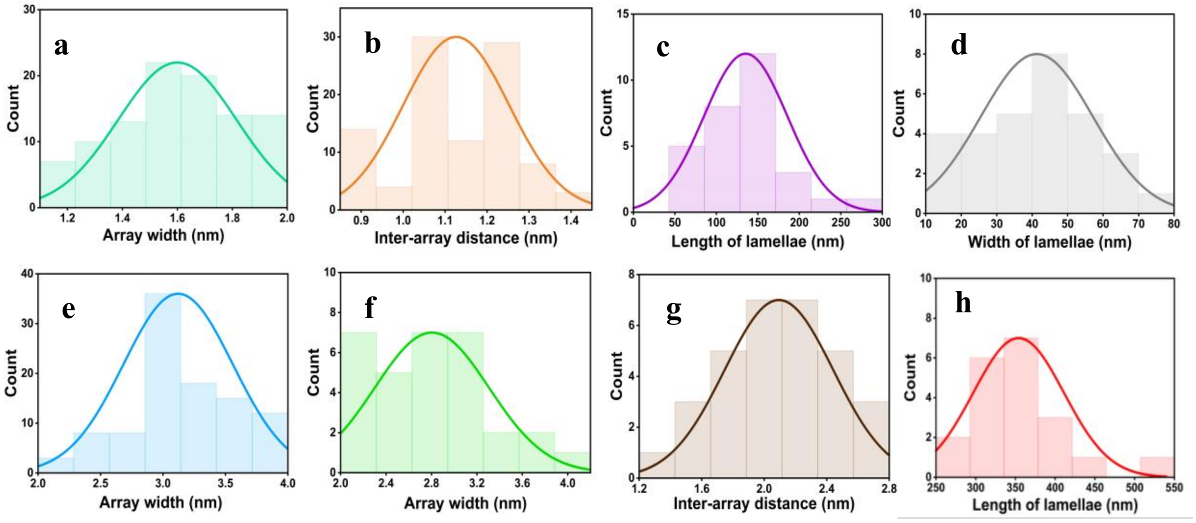

Supplementary Figure 16: Calculated spatial dimensions of 3K coassemblies. Histograms showing size distribution for various 3K coassemblies. a-d) 3K in PBS, e) 3K in LPS, f-h) 3K in *E. coli*. Values were determined from NS-TEM images analyzed with ImageJ software. Distance parameters and the number of objects/particles calculated for each parameter is summarized in Supplementary Table 4.

Supplementary Table 4: Spatial dimensions of 3K coassemblies. Values were calculated from parameters determined based on NS-TEM images using the ImageJ software. The data are represented as mean ± SD where the number of objects analysed are given.

Coassembly	Parameter	No. of objects	Distance (nm)
3K-PBS	Array width	100	1.60 ± 0.22
3K-PBS	Inter-array distance	100	1.13 ± 0.13
3K-PBS	Lamella length	30	135 ± 50.2
3K-PBS	Lamella width	30	41.3 ± 15.8
3K-LPS*	Array width	100	3.12 ± 0.44
3K in *E. coli*	Array width	30	2.82 ± 0.50
3K in *E. coli*	Inter-array distance	30	2.11 ± 0.34
3K in *E. coli*	Lamella length	20	345 ± 29.5
3K in *E. coli*	Lamella width	60	28.7 ± 9.38

*only this parameter could be calculated for the 3K-LPS system due to the highly curved nature of the coassembly

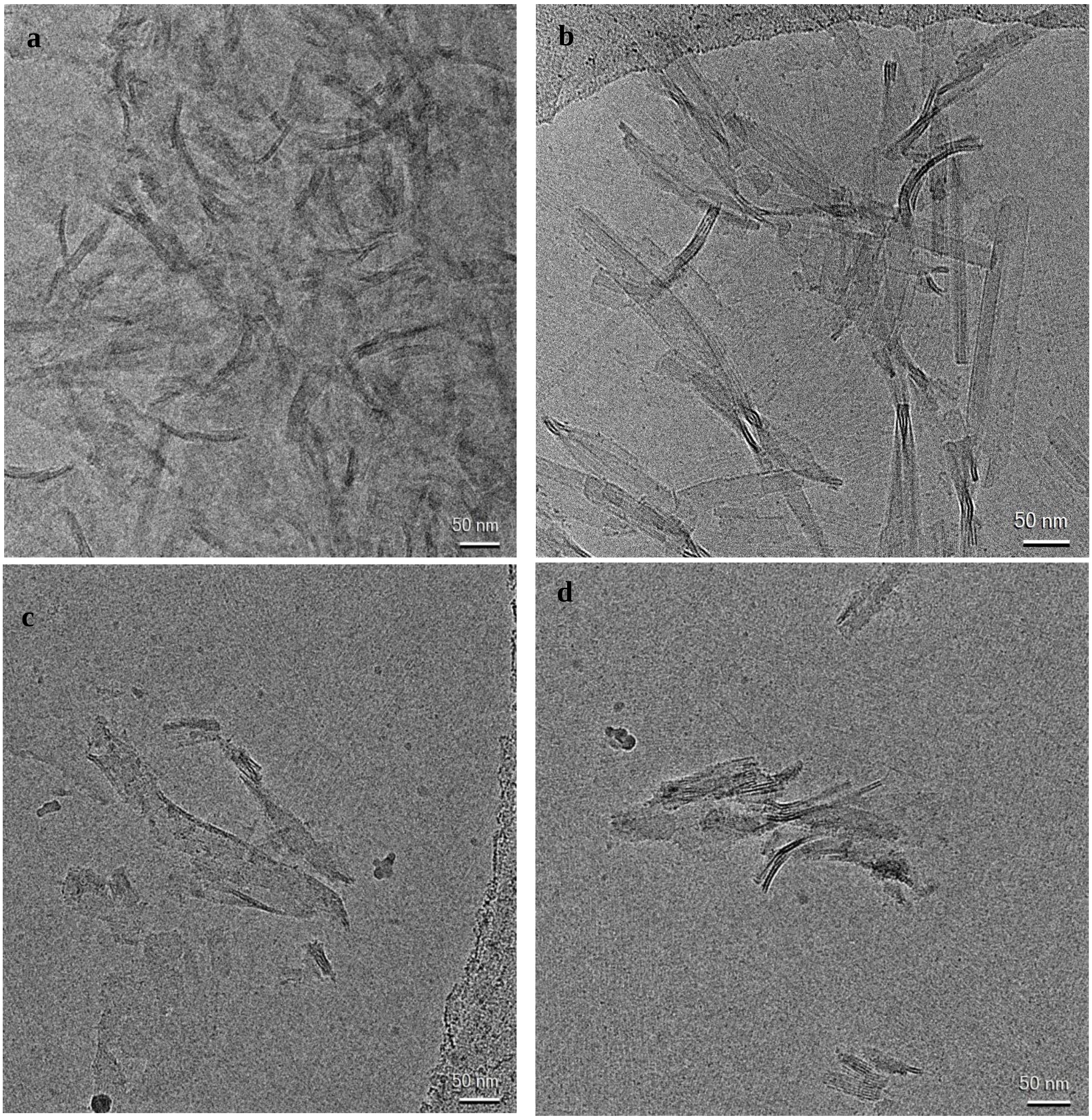

Supplementary Figure 17: Cryo-EM images of 3K (a-d) validate the striped lamella morphology in solution. Samples were prepared at 125 μM peptide in PBS. The cryo-EM morphology highly resembles the characteristic striped sheet morphology observed in NS-TEM images (Fig.1d).

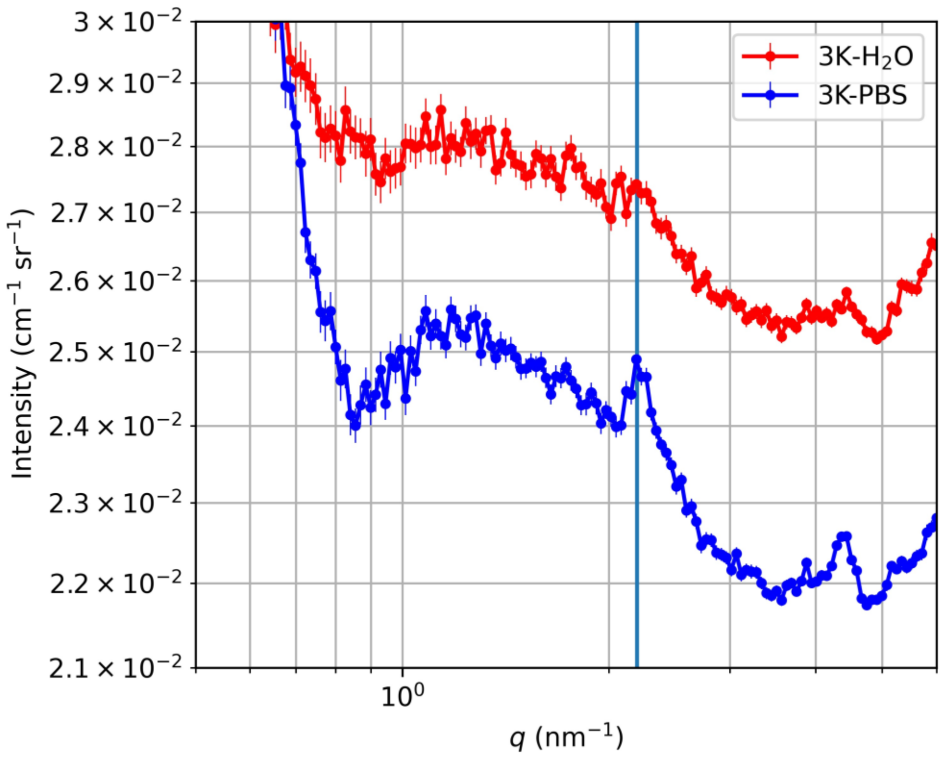

Supplementary Figure 18: Periodic repeat distance determined from SAXS scattering pattern. The SAXS curve of 3K was measured at 10 mM 3K concentration in water and PBS. For PBS, two orders of equidistant peaks appeared, hinting at a regular periodic ordering of scattering units. The position of the first peak was determined by fitting to a Lorentzian function using an orthogonal distance regression (least squares) algorithm, yielding 2.197 ± 0.005 nm^{-1}, which corresponds to a 2.860 ± 0.006 nm periodic repeat distance using the Bragg's equation. In water, a similar peak is visible, at smaller q, corresponding to a larger periodic distance of ~ 3.2 nm. However, no second harmonic is observable, suggesting that the periodicity has a shorter extent in space (i.e. not so long-range order) than found in the PBS solution. Error bars are displayed as lines.

Supplementary Table 5: Essential SAS data acquisition, sample details, data analysis, modelling fitting and software used.

(*a*) Sample details		
	Lamellin-3K in water	Lamellin-3K in PBS
Organism	N/A (synthetic)	N/A (synthetic)
Source (Catalogue No. or reference)	N/A	N/A
Description: sequence (including Uniprot ID + uncleaved tags), bound ligands/modifications, *etc.*	N/A	N/A
Extinction coefficient ε (wavelength and units)	N/A	N/A
Partial specific volume $\bar{\upsilon}$ (cm^3 g^{-1})	N/A	N/A
Mean solute and solvent scattering length densities and mean scattering contrast $\Delta\bar{\rho}$ (cm^{-2})	N/A	N/A
Molecular mass *M* from chemical composition (Da)	N/A	N/A
For SEC-SAS, loading volume/concentration, (mg mL^{-1}) injection volume (μL), flow rate (ml min^{-1})	N/A	N/A
Concentration (range/values) measured and method	N/A	N/A
Solvent composition and source	water	PBS

(*b*) SAS data collection parameters

Source, instrument and description or reference: CREDO, general-purpose 3-pinhole collimated SAXS camera.[18] X-ray source is a GeniX^{3D} Cu ULD integrated beam delivery system.
Wavelength (Å): 1.542 Å.

Beam geometry (size, sample-to-detector distance): circular, 1.3 mm maximum diameter at the sample position, 411 mm sample-to-detector distance.
q-measurement range (Å^{-1} or nm^{-1}): 0.24 nm^{-1} to 6.8 nm^{-1}.
Absolute scaling method: using a pre-calibrated glassy carbon specimen, courtesy of Jan Ilavsky (APS, Chicago, US).
Basis for normalization to constant counts: exposure time.
Method for monitoring radiation damage, X-ray dose where relevant: the final scattering patterns were averaged from multiple, 5 minute-long exposures. Before averaging, pairwise comparison of all distinct exposures was done. Apart from outliers (due to external radiation and cosmic events), no systematic trend was observed throughout the whole experiment.
Exposure time, number of exposures: 14 hours 20 minutes (172 exposures) for the sample in water, 18 hours 45 minutes (225 exposures) for the sample in PBS.
Sample configuration including path length and flow rate where relevant: samples were situated in borosilicate glass capillaries of 1.5 mm outer diameter and 0.01 mm nominal wall thickness. The whole experiment has been carried out at a single point along the capillary.
Sample temperature (°C): 25°C.

(*c*) Software employed for SAS data reduction, analysis and interpretation

SAS data reduction to sample-solvent scattering, and extrapolation, merging, desmearing *etc*. as relevant: External ("dark image") and internal ("empty beam") backgrounds have been subtracted from each scattering pattern. Counts were normalized to unit exposure time and divided by the transmission of the sample. Images were corrected for detector flatness and azimuthally averaged, yielding the scattering curves. All data reduction steps were carried out using the standard data reduction routine implemented in the instrument control software of the CREDO instrument.
Calculation of ε from sequence: N/A.
Calculation of $\Delta\overline{\rho}$ and $\overline{\upsilon}$ values from chemical composition: N/A.
Basic analyses: Guinier, $P(r)$, scattering particle volume (*e.g.* Porod volume V_P or volume of correlation V_c): none of the above.
Shape/bead modelling: N/A.
Atomic structure modelling (homology, rigid body, ensemble): N/A.
Modelling of missing sequence from PDB files: N/A.
Molecular graphics: N/A.

(*d*) Structural parameters

Guinier Analysis	Lamellin-3K in water	Lamellin-3K in PBS
$I(0)$ (cm^{-1})	N/A	N/A
R_g (Å)	N/A	N/A
q-range (Å^{-1})	N/A	N/A
Quality-of-fit parameter (with definition)	N/A	N/A
M from $I(0)$ (ratio to expected value)	N/A	N/A
**$P(r)$ analysis**	**Lamellin-3K in water**	**Lamellin-3K in PBS**
$I(0)$ (cm^{-1})	N/A	N/A
R_g (Å)	N/A	N/A
d_{max} (Å)	N/A	N/A
q-range (Å^{-1})	N/A	N/A
Quality-of-fit parameter (with definition)	N/A	N/A
M from $I(0)$ (ratio to expected value)	N/A	N/A
Volume (*e.g.* V_P and/or V_c)	N/A	N/A

(*e*) Shape modelling results (a complete panel for each method)

	Lamellin-3K in water	Lamellin-3K in PBS
q-range for fitting	N/A	N/A
Symmetry/anisotropy assumptions	N/A	N/A
Ambiguity measure(s) with definitions	N/A	N/A
χ^2 value/range	N/A	N/A
P value, any other quality-of-fit parameters	N/A	N/A
Adjustable parameters in the model fit	N/A	N/A
Model volume and/or M estimate	N/A	N/A
Model precision/resolution	N/A	N/A

For multiple phase shape models, R_g values and relative phase volumes	N/A	N/A
(*f*) Atomistic modelling		
	Lamellin-3K in water	Lamellin-3K in PBS
Method	N/A	N/A
q-range for fitting	N/A	N/A
Symmetry assumptions	N/A	N/A
Any measures of model precision	N/A	N/A
χ^2 value/range	N/A	N/A
P value, any other quality-of-fit parameters	N/A	N/A
Adjustable parameters in the model fit	N/A	N/A
Relevant output parameters (*e.g.* predicted R_g/d_{max} values, weights for multi-state models, *etc.*)	N/A	N/A
Domain/subunit coordinates and contacts, regions of presumed flexibility as appropriate	N/A	N/A
(*g*) Data and model deposition IDs		
	Lamellin-3K in water	Lamellin-3K in PBS
	N/A	N/A

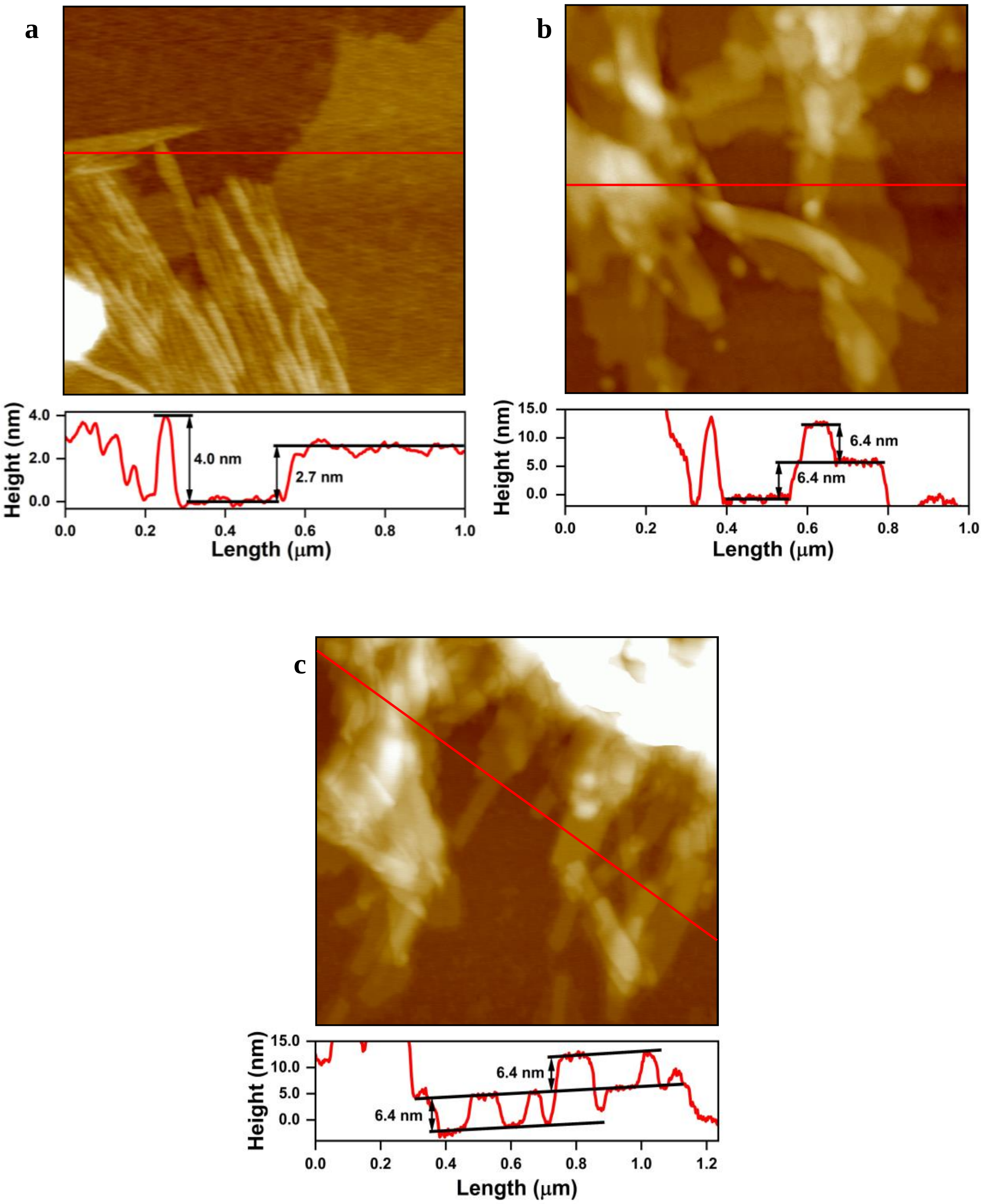

Supplementary Figure 19: AFM images of 3K-phosphate coassemblies reveal concentration dependent morphology variants. For AFM imaging, dried droplet were prepared from 3K dissolved at a) 12.5 µM, b) 125 µM, or c) 1250 µM, while keeping a 3K-to-PBS ratio of 1:4. Height profiles along the marked red line are shown below the images (1 µm x 1 µm). At 12.5 µM 3K, a compact, continuous layer of d = 2.67 ± 0.27 nm (n=26 objects measured) height was observed. At 125 µM peptide, the d = 2.67 ± 0.27 nm thick monolayer was less pronounced, whereas at 1250 µM, only faint traces of thick, loose, non-continuous layer could be detected. A characteristic height difference of d = 6.43 ± 0.48 nm (n=17 objects measured) was also revealed. Particularly, at 125 µM 3K, the dominant objects were thick, overlapping, elongated, lamellar structures, which resemble well the 3K morphology observed in NS-TEM images at this concentration range. Partially aligned and intertwined individual fibrils were also visible with a typical height of 4.0 nm (a, bottom, left), and some dot-like structures were also detected (b, bottom, middle/left). The data are represented as mean ± SD.

a

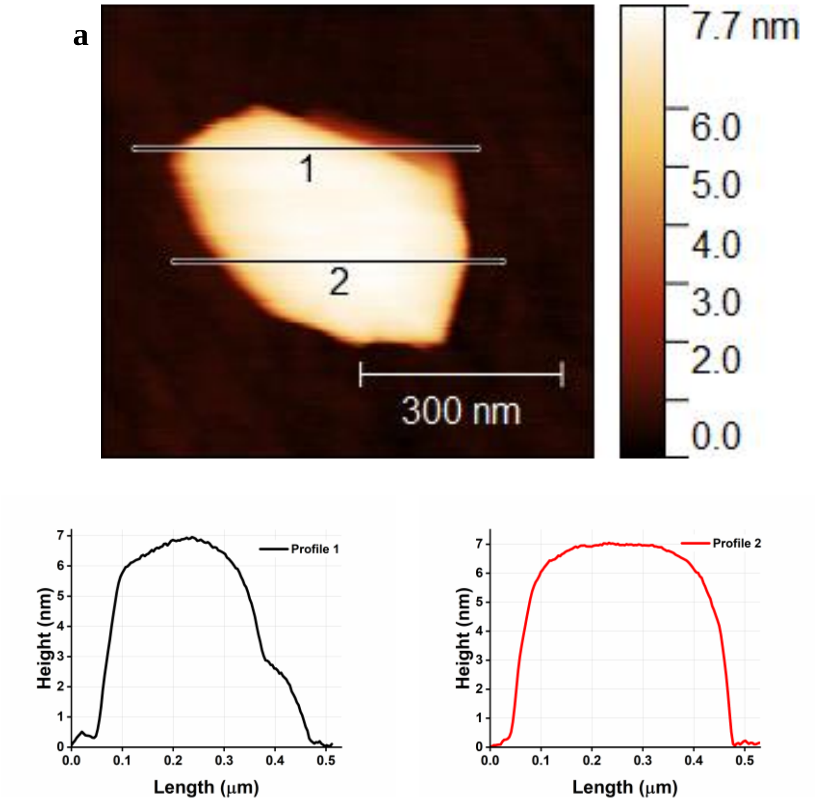
1
2
300 nm
7.7 nm
6.0
5.0
4.0
3.0
2.0
0.0
Profile 1
Height (nm)
Length (µm)
Profile 2
Height (nm)
Length (µm)

b

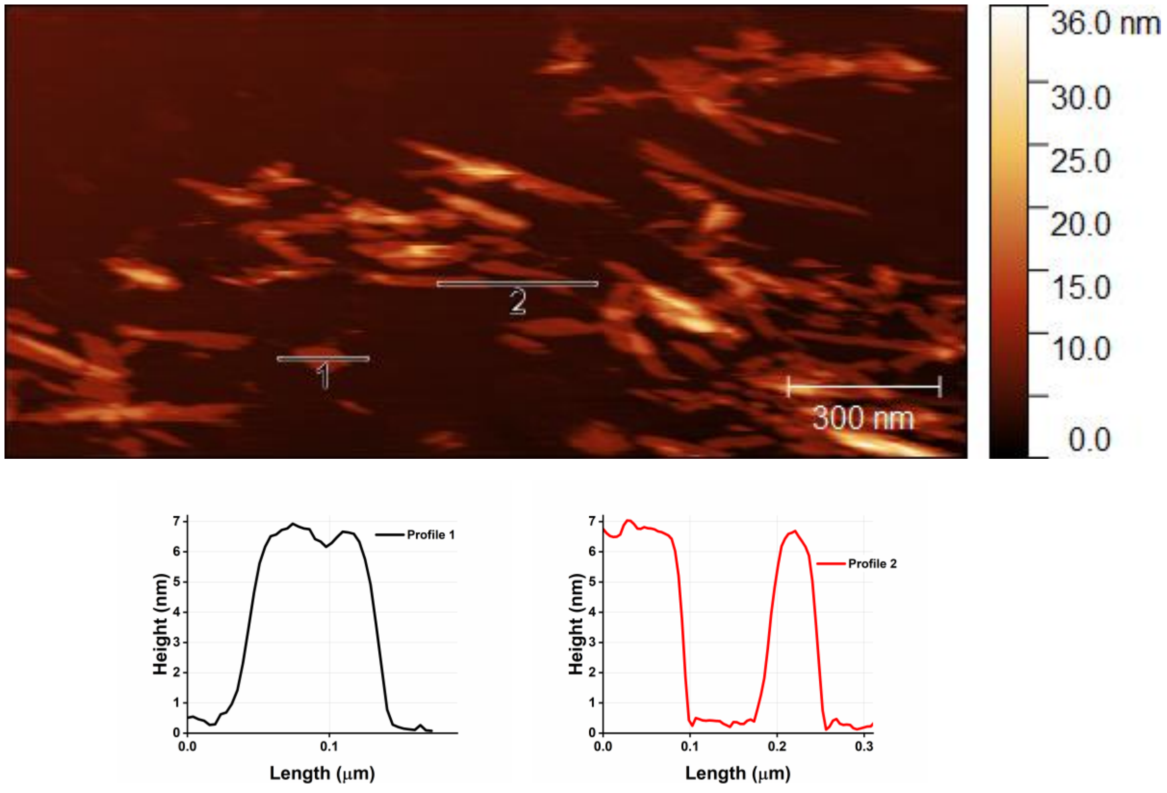
1
2
300 nm
36.0 nm
30.0
25.0
20.0
15.0
10.0
0.0
Profile 1
Height (nm)
Length (µm)
Profile 2
Height (nm)
Length (µm)

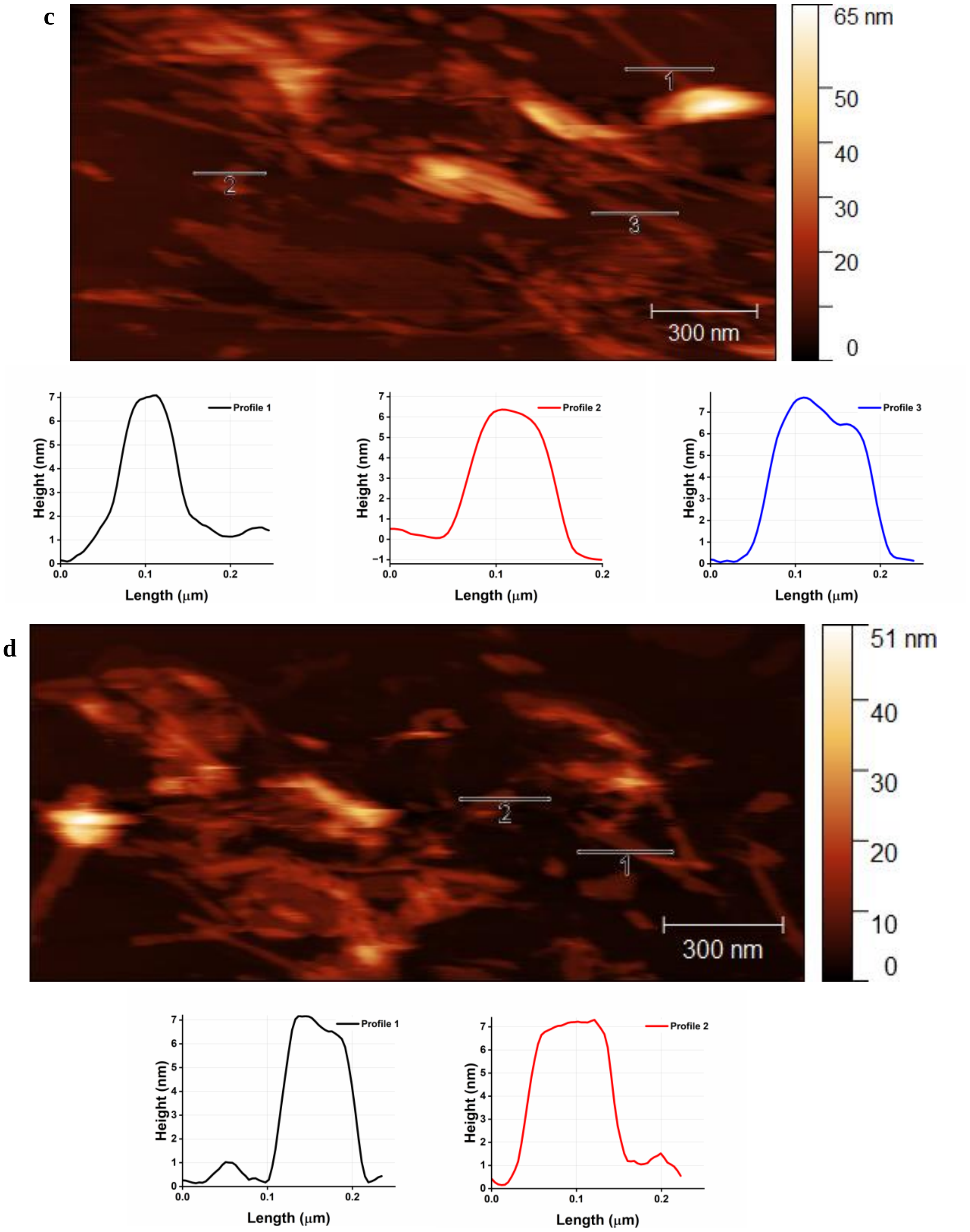

Supplementary Figure 20: liquid AFM images of 3K-PBS. 3K (125 μM) was dissolved in PBS (500 μM). Freshly cleaved muscovite mica was used as a substrate. 50 μL of the 3K solution was deposited onto the mica disc and left to adsorb for 5 min before imaging. Topography data recorded at four randomly selected locations are shown in the images (a, b, c, d). Height profiles along the marked lines are shown below the images. The surface morphology appears similar to those found in dry AFM and EM images. The lamellar structures had a characteristic thickness of 6.24 ± 0.91 nm (n=50 objects measured) with a confidence interval of 0.3 nm (95% confidence level, determined from 50 height profiles). The data are represented as mean ± SD.

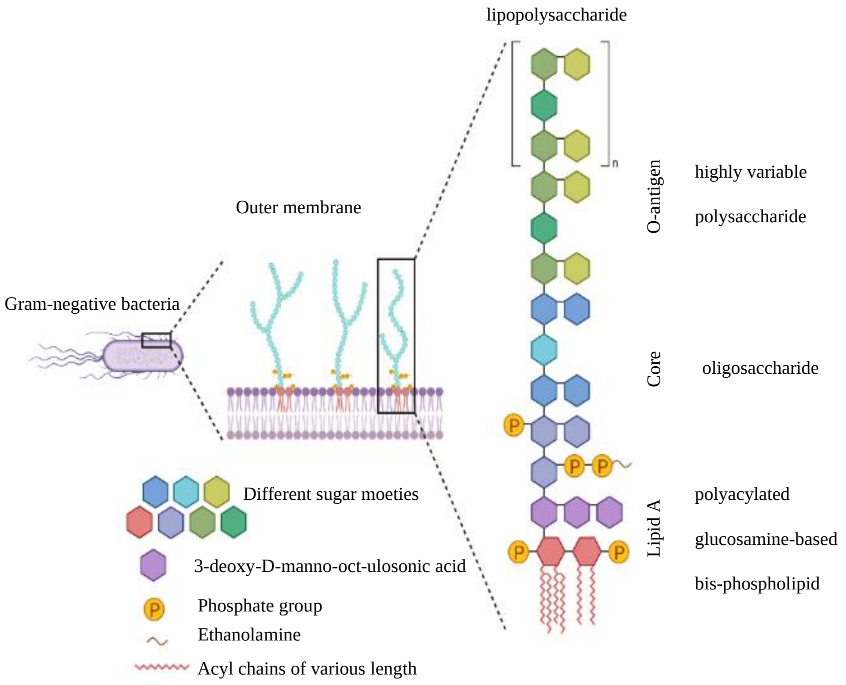

Supplementary Figure 21: Schematic representation of bacterial lipopolysaccharides. LPS is the most abundant lipid component in the outer leaflet of the outer membrane of Gram-negative bacteria. The complex LPS molecule can be divided in three main structural parts. The conserved lipid A part contains several acyl chains, which anchors the molecule into the bacterial membrane, and some sugar moieties decorated with several phosphate groups. The middle part is a less conserved core oligosaccharide region that links lipid A to the O-antigen. The O-antigen is the outermost part, highly variable in length and sugar composition. In some strains, the O-antigen is missing, which converts the smooth surface (i.e., with the O-antigen) to rough. Figure created with Biorender.com

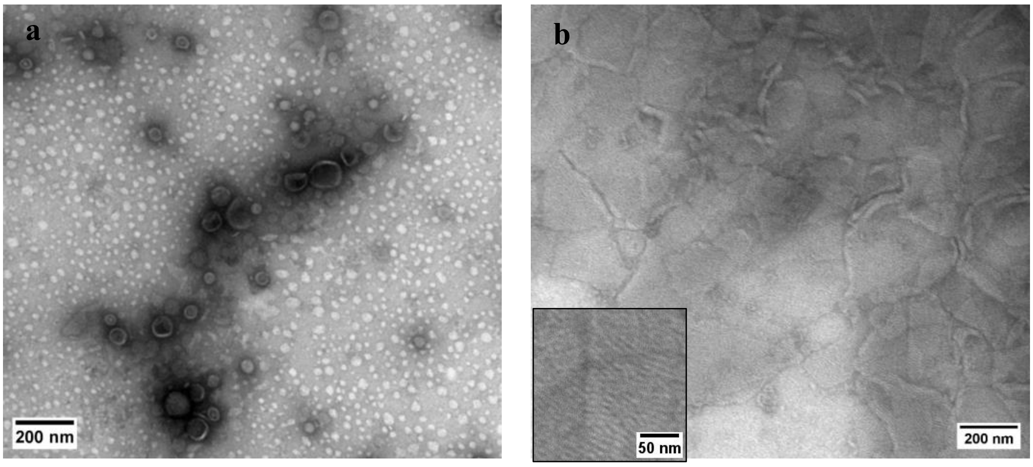

Supplementary Figure 22: NS-TEM image of (a) LPS as control and (b) LPS treated with 3K showing curved striped supramolecular morphology.

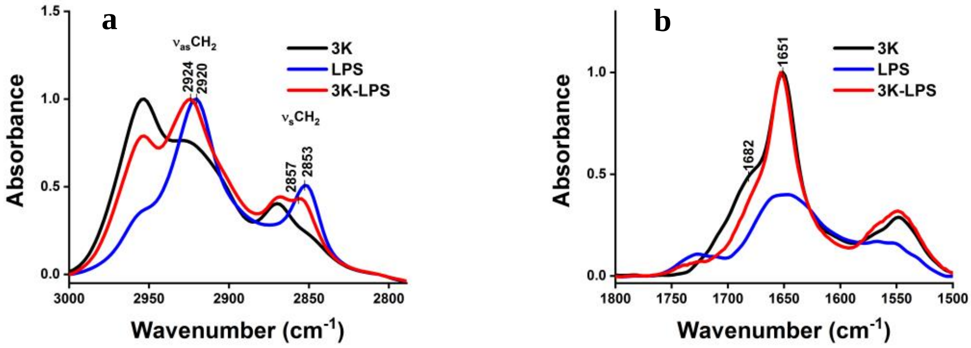

Supplementary Figure 23: ATR-IR spectra report on insertion of aligned peptides between the LPS chains in the 3K-LPS coassembly. a) Methylene vibration region characteristic of order and packing of lipid acyl chains. The marked shift in both the anti-symmetric and symmetric stretching vibration bands (from 2920 cm^{-1} to 2924 cm^{-1} for $\nu_{as}CH_2$ and from 2853 cm^{-1} to 2857 cm^{-1} for $\nu_{s}CH_2$) of the LPS methylene moieties upon addition of 3K, clearly indicates that interaction with 3K significantly perturbs the packing of lipid A chains. b) Peptide amide I and amide II regions. The strong IR signal at 1651 cm^{-1} can be assigned to a dominant random coil conformation, while the satellite signal at 1682 cm^{-1} to turn structures. In the 3K-LPS coassembly, turns are less favoured so that the amide units along the sequence adopt a more uniform conformation likely resulting in an extended backbone compared to that in the free peptide.

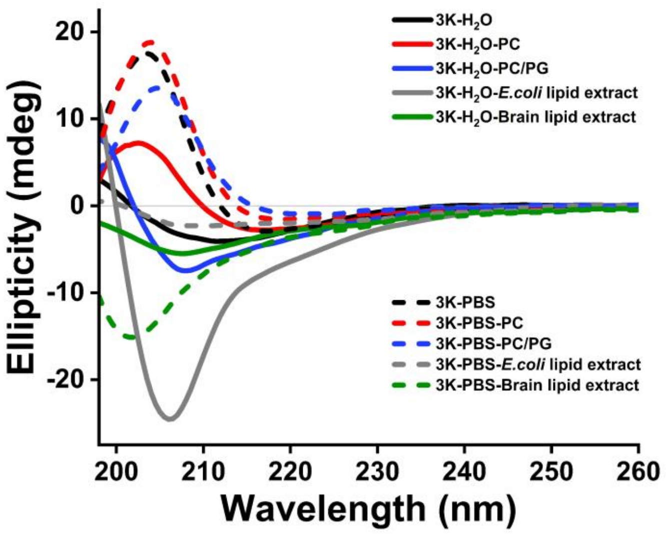

Supplementary Figure 24: CD spectral pattern suggests minor peptide conformational changes upon binding to model vesicles. Formation of 3K-phosphate coassemblies was tested in more complex, but biologically relevant phosphate environments. Artificial model membranes such as liposomes prepared from i) the zwitterionic DOPC, ii) DOPC/DOPG (8:2) iii) *E. coli* Polar Lipid Extract and iv) Brain Total Lipid Extract (from porcine brain) were employed. In water, only the interaction with the PC membrane showed a moderate change towards CD of 3K-PBS system, whereas all other tested systems showed clearly distinct CD curves from that. Here the vesicles from *E. coli* polar lipid extract indicate some formation of order, though the CD spectrum does not resemble to the 3K lamellar morphologies. In PBS, besides some intensity variation, the spectral pattern of 3K-PBS was preserved in the presence of PC and PC/PG liposomes. These results indicate that potential lipid binding does not affect markedly the solution-phase form of the peptides adopted prior interaction and that none of the environments induce formation of lamellar morphology akin to phosphates in PBS solution.

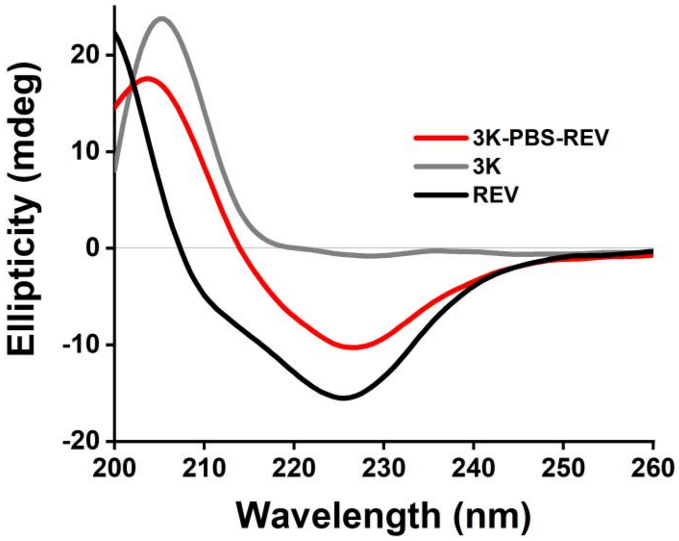

Supplementary Figure 25: CD spectral pattern of 3K in the presence of extracellular vesicles. Extracellular vesicles derived from human red blood cells (REVs) were used as a complex model membrane system to test its effect on co-assembly formation of 3K. In PBS, the preservation of the 3K-PBS morphology can be observed indicating that no significant interaction occurs between lamellin-3K and the REV vesicles. Note that for this experiment only PBS buffer was used, as in pure water strong REV lysis occurs. Moreover, the contribution of the large number of proteins present in the REVs (> 121, based on proteomic results[19]) can be attributed to the broad negative peak at ~ 225 nm.

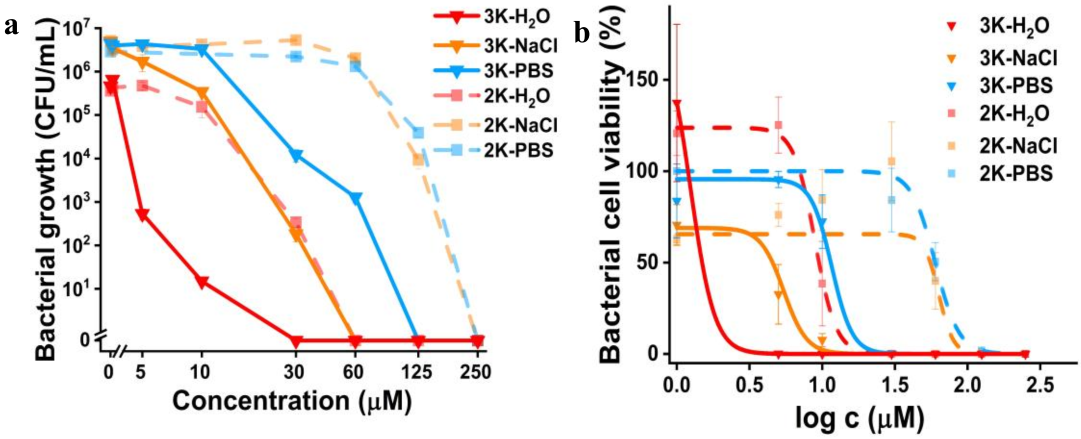

Supplementary Figure 26: Antibacterial activity of 2K and 3K in the presence and absence of phosphate ions. a) Growth inhibition curves. Exponentially growing *E. coli* BL21 shaker cultures were exposed to the peptides at 37 °C in various media for 120 min. Treatments were performed in three biological replicates, each of which in three technical replicates. Data represent mean and error bars represent SEM. Data points are connected to guide the eye. b) Viability curves calculated from growth inhibition data in (a). Smooth lines are sigmoid fits to the data points.

Supplementary Table 6: Antimicrobial activity of 2K and 3K in various media.

Compound	Medium	*IC_{80} [μM]	IC_{50} [μM]	IC_{20} [μM]
2K	Water	11.2 ± 0.373	8.91 ± 0.364	7.06 ± 0.343
	PBS	77.7 ± 3.93	59.8 ± 3.62	46.1 ± 4.07
	NaCl	74.2 ± 10.8	63.3 ± 11.6	54.0 ± 12.2
3K	Water	1.62 ± 5.6 E-4	1.24 ± 5.0 E-4	0.957 ± 4.35 E-4
	PBS	14.6 ± 1.46	11.6 ± 1.48	9.27 ± 1.45
	NaCl	7.00 ± 1.02	5.44 ± 0.91	4.22 ± 0.82

* IC values are calculated from sigmoid fits to the viability vs. log(c) data points.
All errors are standard errors.

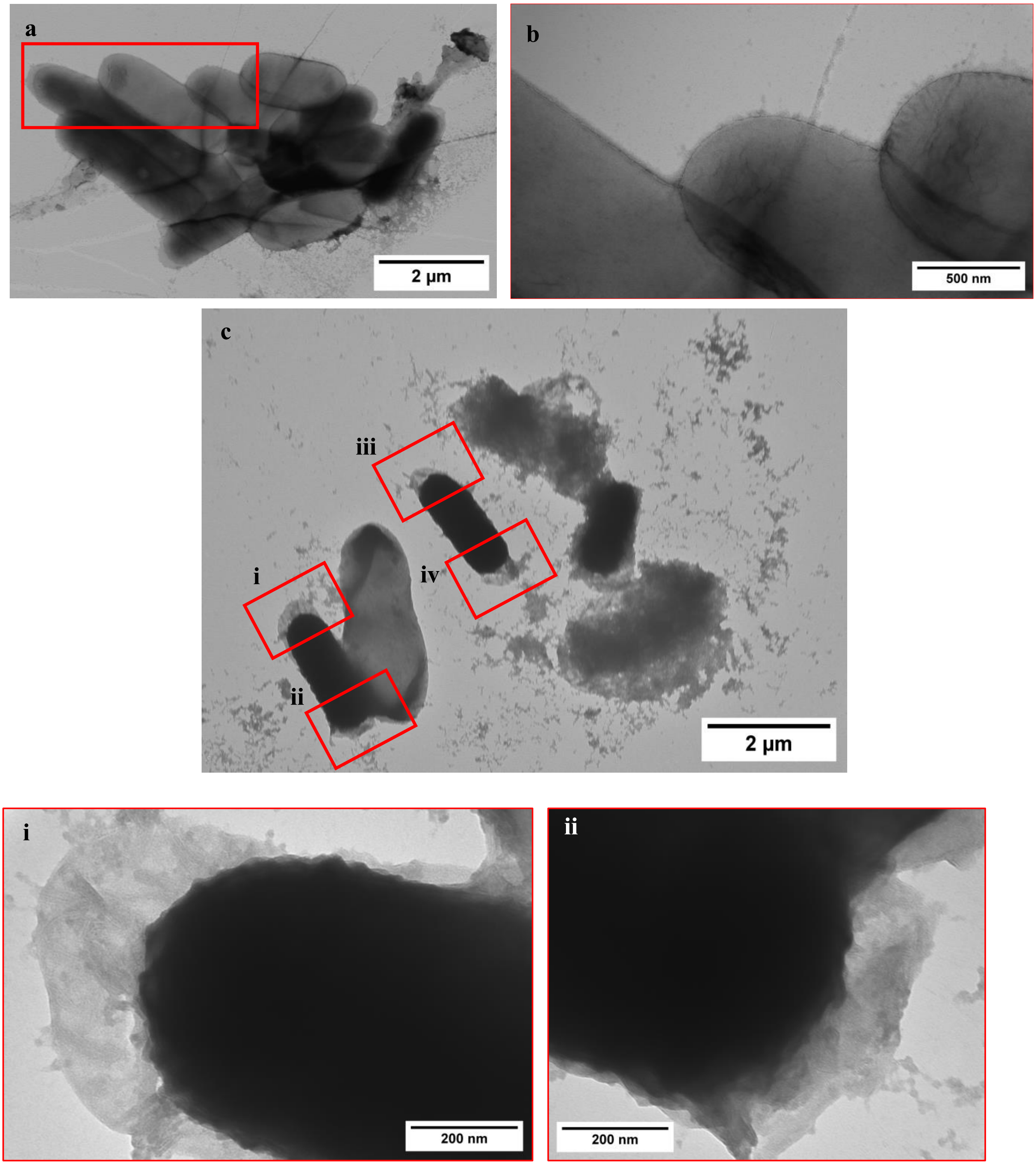
a
2 μm
b
500 nm
c
iii
i
iv
ii
2 μm
i
200 nm
ii
200 nm

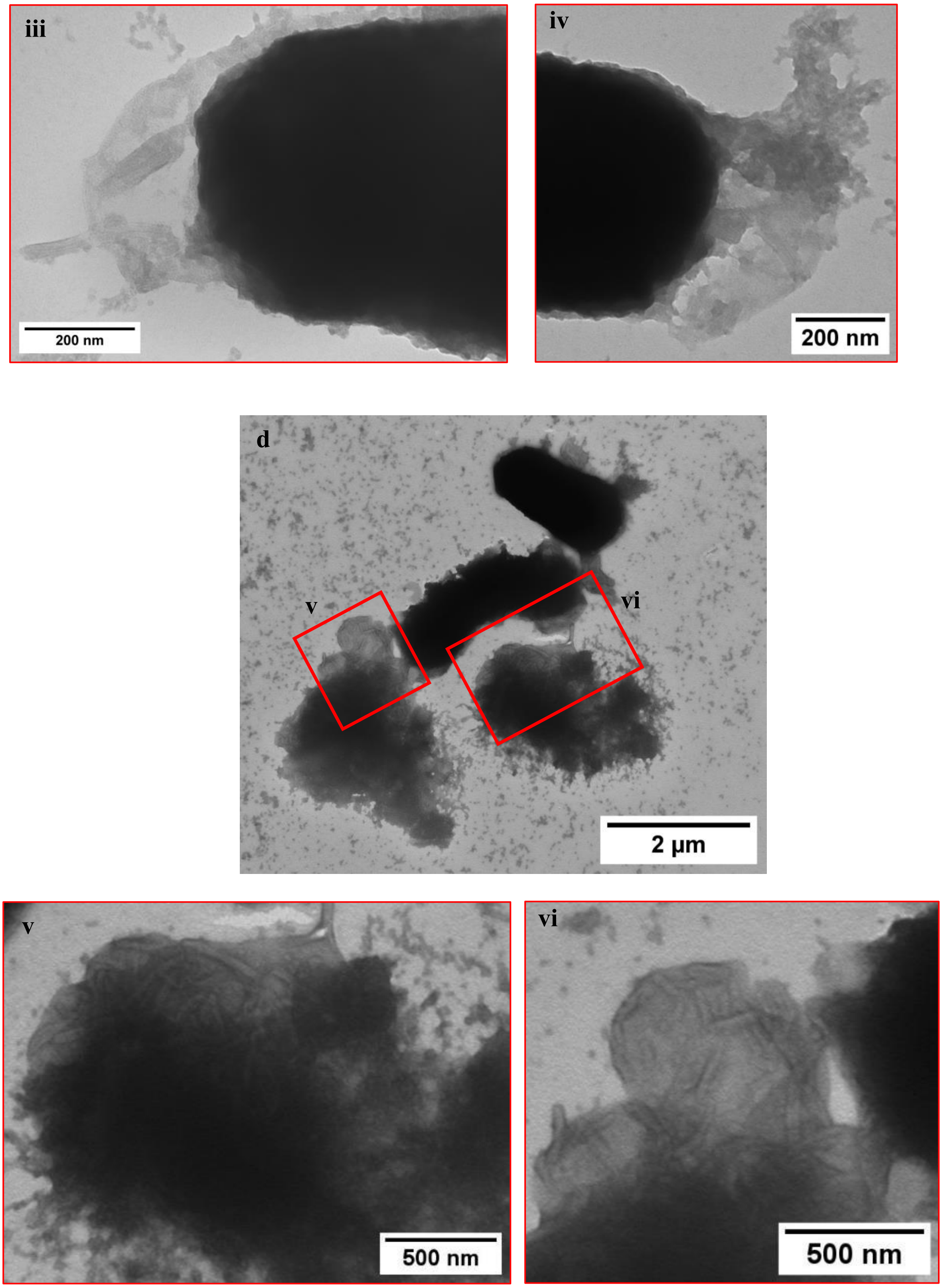

Supplementary Figure 27: Electron micrographs of 3K treated *E. coli* cells show damaged cells and leakage. Bacterial cells were treated with 5 µM 3K in water for 120 min and negative-stained with uranyl acetate. a,b) NS-TEM image of untreated bacteria show intact cell wall integrity. c) 3K assembly formation perturbs bacterial membrane integrity allowing leakage of the intracellular liquid from damaged cells. (i-iv) Magnified cell parts highlight the toxic effect. (d) Intensive 3K assembly formation can be observed in sub-membrane regions, which results in highly perturbed cell surface integrity. (v-vi) Magnified cell parts highlight the 3K assembly formation.

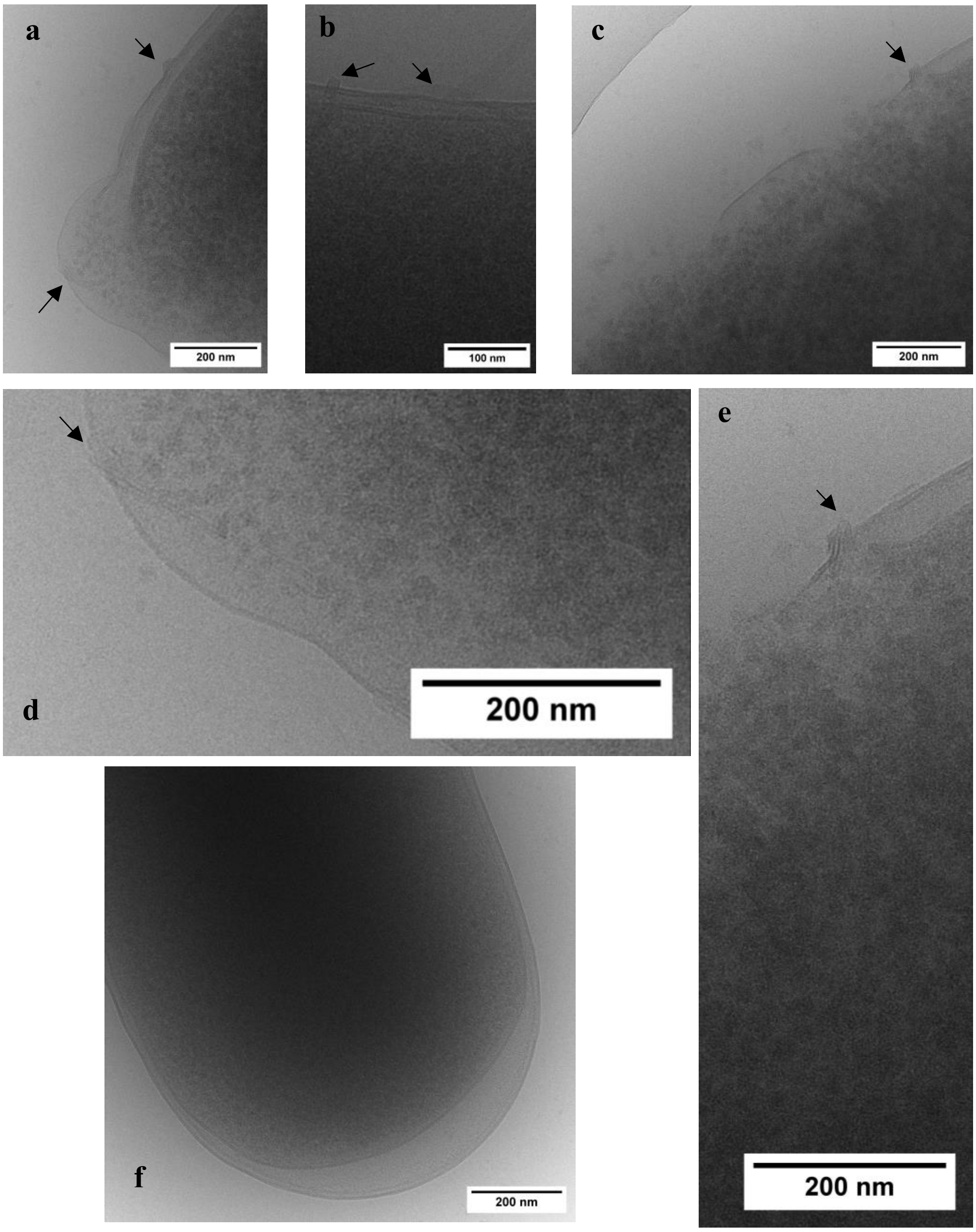

Supplementary Figure 28: Cryo-EM of 3K-treated *E. coli* cells. Bacterial cells were treated with 5 µM 3K in water for 40 min (a-e). (d) Magnification from (a). (e) Magnification from (c). (f) Image of untreated control *E. coli*.

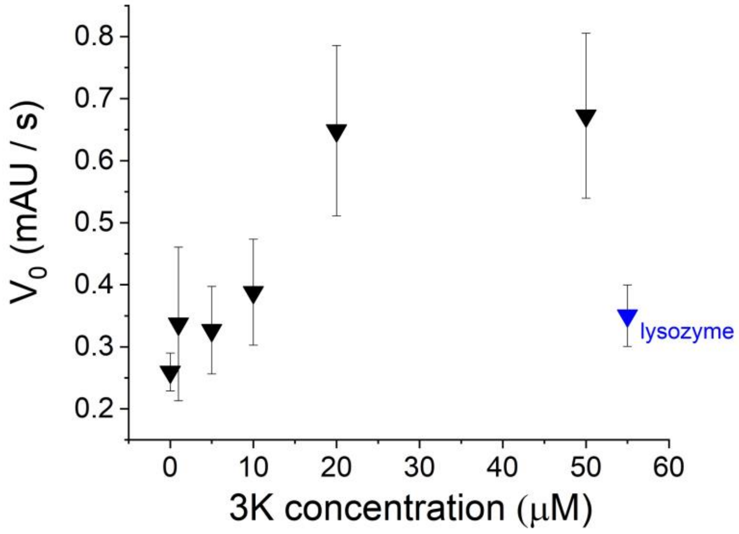

Supplementary Figure 29: Leakage Assays of 3K treated *E. coli* cells. Changes in outer membrane permeability induced by 3K. Initial velocities of nitrocefin degradation following a 20-minute incubation of bacterial cells with 3K in PBS at 0, 1, 5, 10, 20 50 µM concentrations. Lysozyme was used as a positive control as it degrades the bacterial cell wall and promotes the release of intracellular contents including β-lactamase. Data points are the average of four biological replicates. Error bars indicate standard deviation.

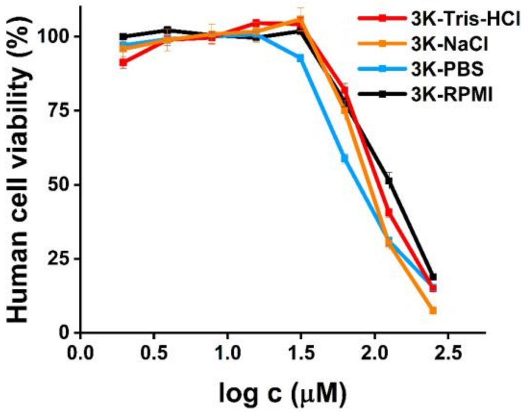

Supplementary Figure 30: Cytotoxic activity of 3K in presence and absence of phosphate ions. Human MonoMac-6 cells were treated with the 3K for 120 min in various media. Each point represents the mean of four parallel experiments and error bars represent SEM. The viability percentage was calculated relative to the untreated control. RPMI (or RPMI-1640) is the culture media used for MonoMac-6 cells.

Supplementary Table 7: Cytotoxic activity of 3K in various media. The data are represented as mean ± SEM (n=4 independent biological samples).

**Media**	**IC_{50} [μM] ± SEM**
Tris-HCl	112.2 ± 8.4
NaCl	94.44 ± 7.75
PBS	83.60 ± 5.55
RPMI	126 ± 7.1

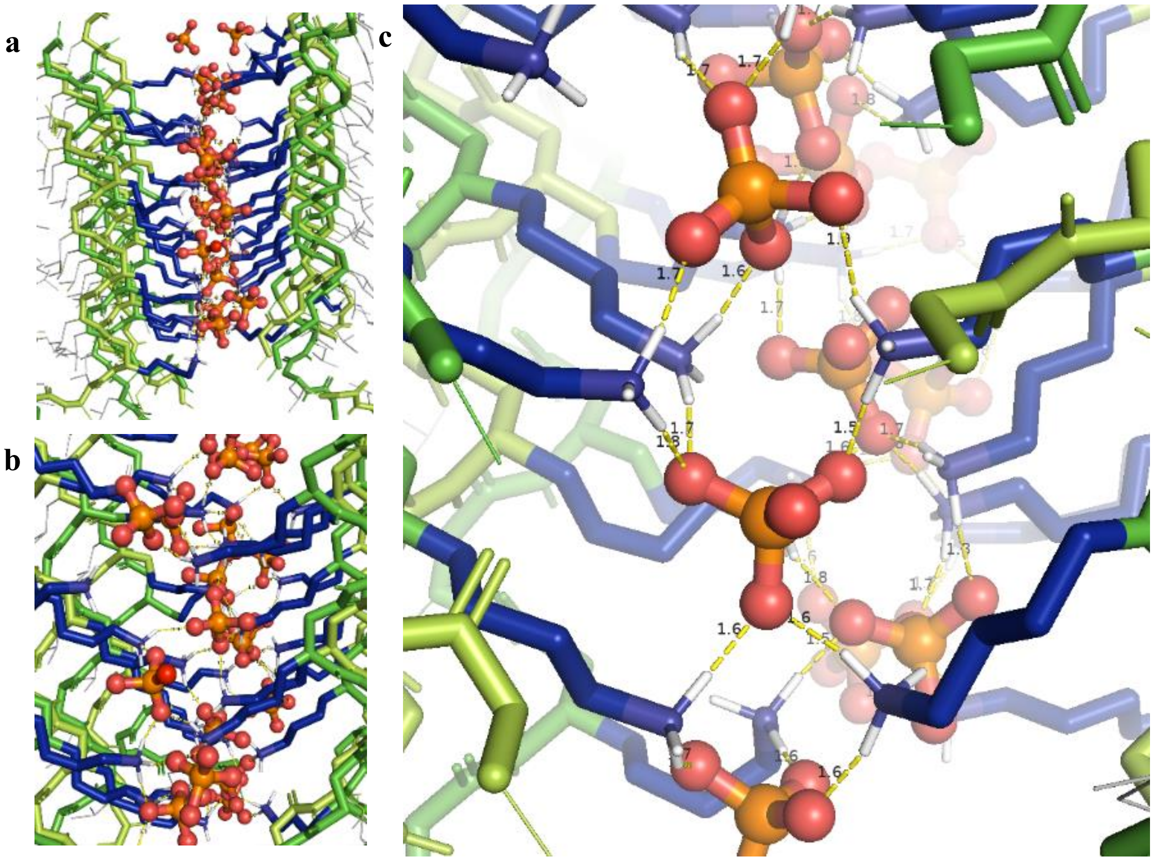

Supplementary Figure 31: The salt-bridge network within the 3K-phosphate coassembly assessed from MD simulation. a) Representative snapshot of the 24-mer showing two peptide layers interconnected by a phosphate layer. b) Salt-bridges formed between lysine side chains and phosphate groups. c) Multiple hydrogen bonds formed between the charged side chain NH_3^+ groups and the phosphate oxygens. Peptide backbones and lysine side chains of 3K are displayed as green and blue sticks, respectively, whereas leucine side chains are displayed as grey lines. Phosphate ions are as ball and sticks, where oxygen atoms are red, and phosphor atoms are orange. Salt bridges are marked as dashed yellow lines. Distance between the donor and acceptor atoms is given in Ångströms. For clarity, only salt bridges of < 2 Å are labelled, while neither structural water nor phosphate methylene groups are displayed.

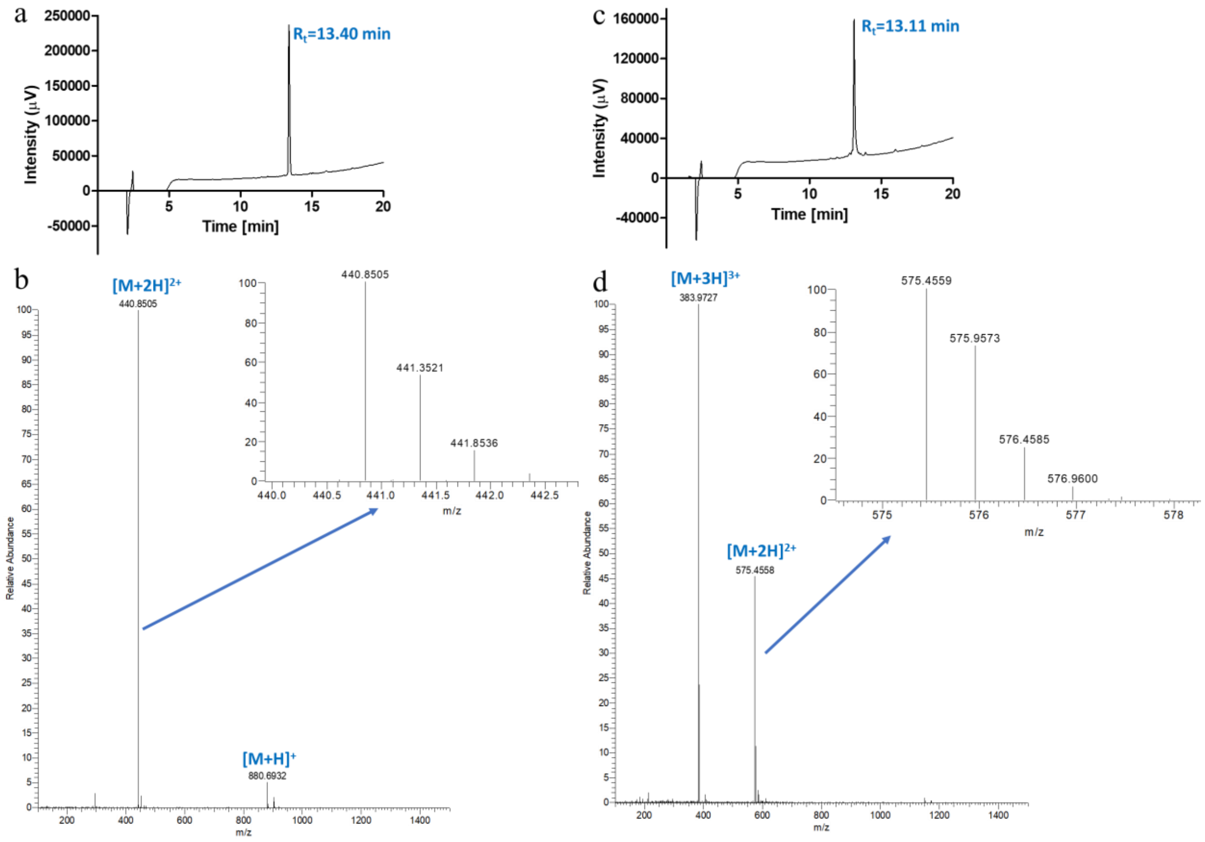

Supplementary Figure 32. RP-HPLC chromatograms (a,c) and HRMS spectra (b,d) of 2K and 3K.
Measured monoisotopic molecular mass for 2K (b) = 879.6864, calculated monoisotopic M_w for $C_{46}H_{89}N_9O_7$ = 879.6885, Δppm = 2.136. Measured monoisotopic molecular mass for 3K (d) = 1148.8972, calculated monoisotopic M_w for $C_{60}H_{116}N_{12}O_9$ = 1148.8988, Δppm = 1.643.

References

1. Lindahl, Abraham, Hess & Spoel, van der. GROMACS Documentation. *GROMACS 2021.3 Man.* 1–623 (2021).

2. Abraham, M. J. *et al.* Gromacs: High performance molecular simulations through multi-level parallelism from laptops to supercomputers. *SoftwareX* **1**–**2**, 19–25 (2015).

3. Wacha, A., Beke-Somfai, T. & Nagy, T. Improved Modeling of Peptidic Foldamers Using a Quantum Chemical Parametrization Based on Torsional Minimum Energy Path Matching. *Chempluschem* **84**, 927–941 (2019).

4. Wacha, A. & Beke-Somfai, T. PmlBeta: A PyMOL extension for building β-amino acid insertions and β-peptide sequences. *SoftwareX* **13**, 100654 (2021).

5. Berendsen, H. J. C., Postma, J. P. M., Van Gunsteren, W. F., Dinola, A. & Haak, J. R. Molecular dynamics with coupling to an external bath. *J. Chem. Phys.* **81**, 3684–3690 (1984).

6. Bussi, G., Donadio, D. & Parrinello, M. Canonical sampling through velocity rescaling. *J. Chem. Phys.* **126**, (2007).

7. Parrinello, M. & Rahman, A. Polymorphic transitions in single crystals: A new molecular dynamics method. *J. Appl. Phys.* **52**, 7182–7190 (1981).

8. Wacha, A., Varga, Z. & Beke-Somfai, T. Comparative Study of Molecular Mechanics Force Fields for β-Peptidic Foldamers: Folding and Self-Association. *J. Chem. Inf. Model.* **63**, 3799–3813 (2023).

9. Szigyártó, I. C. *et al.* Membrane active Janus-oligomers of β3-peptides. *Chem. Sci.* **11**, 6868–6881 (2020).

10. Wacha, A. F. & Lemkul, J. A. charmm2gmx: An Automated Method to Port the CHARMM Additive Force Field to GROMACS. *J. Chem. Inf. Model.* **63**, 4246–4252 (2023).

11. Seebach, D., Abele, S., Gademann, K. & Jaun, B. Pleated sheets and turns of β-peptides with proteinogenic side chains. *Angew. Chemie - Int. Ed.* **38**, 1595–1597 (1999).

12. Pohl, G., Beke-Somfai, T., Csizmadia, I. G. & Perczel, A. Exploiting diverse stereochemistry of β-amino acids: Toward a rational design of sheet-forming β-peptide systems. *Amino Acids* **43**, 735–749 (2012).

13. Pohl, G., Beke, T., Csizmadia, I. G. & Perczel, A. Extended apolar β-peptide foldamers: The role of axis chirality on β-peptide sheet stability. *J. Phys. Chem. B* **114**, 9338–9348 (2010).

14. Martinek, T. A., Tóth, G. K., Vass, E., Hollósi, M. & Fülöp, F. cis-2-aminocyclopentanecarboxylic acid oligomers adopt a sheetlike structure: Switch from helix to nonpolar strand. *Angew. Chemie - Int. Ed.* **41**, 1718–1721 (2002).

15. Barth, A. Infrared spectroscopy of proteins. *Biochim. Biophys. Acta - Bioenerg.* **1767**, 1073–1101 (2007).

16. Hetényi, A., Mándity, I. M., Martinek, T. A., Tóth, G. K. & Fülöp, F. Chain-length-dependent helical motifs and self-association of β-peptides with constrained side chains. *J. Am. Chem. Soc.* **127**, 547–553 (2005).

17. Möhle, K., Günther, R., Thormann, M., Sewald, N. & Hofmann, H. J. Basic conformers in β-peptides. *Biopolymers* **50**, 167–184 (1999).

18. Wacha, A., Varga, Z. & Bóta, A. CREDO: A new general-purpose laboratory instrument for small-angle X-ray scattering. *J. Appl. Crystallogr.* **47**, 1749–1754 (2014).

19. Singh, P. *et al.* Removal and identification of external protein corona members from RBC-derived extracellular vesicles by surface manipulating antimicrobial peptides. *J. Extracell. Biol.* **2**, (2023).
