# Supplementary material for: In situ captured antibacterial action of membrane-incising peptide lamellae
Source: Nat Commun. 2024 Apr 23;15:3424. doi: 10.1038/s41467-024-47708-4 (PMC11039730; doi:10.1038/s41467-024-47708-4)
Supplement: Supplementary file 3 — Reporting Summary [file 41467_2024_47708_MOESM3_ESM.pdf]

nature portfolio

Corresponding author(s): Tamás Beke-Somfai

Last updated by author(s): Feb 29, 2024

Reporting Summary

Nature Portfolio wishes to improve the reproducibility of the work that we publish. This form provides structure for consistency and transparency in reporting. For further information on Nature Portfolio policies, see our Editorial Policies and the Editorial Policy Checklist.

Please do not complete any field with "not applicable" or n/a. Refer to the help text for what text to use if an item is not relevant to your study.
For final submission: please carefully check your responses for accuracy; you will not be able to make changes later.

Statistics

For all statistical analyses, confirm that the following items are present in the figure legend, table legend, main text, or Methods section.

n/a	Confirmed	
☐	☑	The exact sample size (n) for each experimental group/condition, given as a discrete number and unit of measurement
☐	☑	A statement on whether measurements were taken from distinct samples or whether the same sample was measured repeatedly
☐	☑	The statistical test(s) used AND whether they are one- or two-sided<br>*Only common tests should be described solely by name; describe more complex techniques in the Methods section.*
☐	☑	A description of all covariates tested
☑	☐	A description of any assumptions or corrections, such as tests of normality and adjustment for multiple comparisons
☐	☑	A full description of the statistical parameters including central tendency (e.g. means) or other basic estimates (e.g. regression coefficient) AND variation (e.g. standard deviation) or associated estimates of uncertainty (e.g. confidence intervals)
☑	☐	For null hypothesis testing, the test statistic (e.g. F, t, r) with confidence intervals, effect sizes, degrees of freedom and P value noted<br>*Give P values as exact values whenever suitable.*
☑	☐	For Bayesian analysis, information on the choice of priors and Markov chain Monte Carlo settings
☑	☐	For hierarchical and complex designs, identification of the appropriate level for tests and full reporting of outcomes
☑	☐	Estimates of effect sizes (e.g. Cohen's d, Pearson's r), indicating how they were calculated

Our web collection on statistics for biologists contains articles on many of the points above.

Software and code

Policy information about availability of computer code

Data collection

CD: JASCO J-1500 spectropolarimeter (JASCO, Tokyo, Japan), ATR-FTIR: Varian 2000 FTIR Scimitar spectrometer equipped with a Golden Gate accessory (Varian Inc., Palo Alto, CA). Fluorescence: JASCO FP-8500, NMR: a Bruker Avance III 700 MHz spectrometer (operating at 700.0 MHz for 1H and 176.03 MHz for 13C) equipped with a Prodigy TCI H&F-C/N-D 5 mm probe head with z-gradient, TEM:Morgagni 268D (FEI, The Netherlands), cryo-EM: Talos Arctica (ThermoScientific), AFM: 3100 AFM instrument equipped with a NanoScope IIIa controller (Digital Instruments/Veeco, USA, liquid-AFM: Nanosurf FlexAFM system (Nanosurf, Switzerland), SAXS: CREDO, our in-house pinhole SAXS camera.68,69 Monochromatic Cu Kα X-rays (λ ~ 0.151 nm) are generated using a GeniX3D Cu ULD microfocus X-ray source (Xenocs SA, Sassenage, France). Antimicrobial activity assay: BioTek Synergy Mx plate reader, Leakage assay: CLARIOstar plus plate reader (BMG Labtech), MD Simulations : 2021.6 of the GROMACS software

Data analysis

ATR-FTIR: GRAMS/32 software package (Galactic Inc, USA). NMR:TopSpin by fitting the Stejskal-Tanner equation, SAXS: Scattering patterns were recorded with a Pilatus-300k two-dimensional CMOS hybrid pixel detector (Dectris Ltd, Baden, Switzerland), Cytotoxic activity, Cytotoxic activity: GraphPad Prism software package, EM: ImageJ 1.53t software, spectroscopic techniques: Spectra Manager Version 2.12.00 and OriginPro, Version 2023. OriginLab Corporation, Northampton, MA, USA.

For manuscripts utilizing custom algorithms or software that are central to the research but not yet described in published literature, software must be made available to editors and reviewers. We strongly encourage code deposition in a community repository (e.g. GitHub). See the Nature Portfolio guidelines for submitting code & software for further information.

Data

Policy information about availability of data

All manuscripts must include a data availability statement. This statement should provide the following information, where applicable:

- Accession codes, unique identifiers, or web links for publicly available datasets
- A description of any restrictions on data availability
- For clinical datasets or third party data, please ensure that the statement adheres to our policy

All the data pertaining to this statement are available in this paper and the Supplementary Information.
The MD simulation files can be found at https://doi.org/10.5281/zenodo.8363528 and the NMR chemical shifts are deposited to BMRB with entry numbers 51854 and 51856

Research involving human participants, their data, or biological material

Policy information about studies with human participants or human data. See also policy information about sex, gender (identity/presentation), and sexual orientation and race, ethnicity and racism.

Reporting on sex and gender	For this study, red blood cell-derived extracellular vesicles (REVs) were isolated from healthy donors. The sex and gender of the humans are not relevant for REV - 3K interaction studies.
Reporting on race, ethnicity, or other socially relevant groupings	For this study, red blood cell-derived extracellular vesicles (REVs) were isolated from healthy donors. T he sex and gender of the humans are not relevant for REV - 3K interaction studies.
Population characteristics	For this study, red blood cell-derived extracellular vesicles (REVs) were isolated from healthy donors. T he sex and gender of the humans are not relevant for REV - 3K interaction studies.
Recruitment	The selection of donors was voluntary
Ethics oversight	The usage of human blood samples was approved by the Scientific and research Ethics Committee of the Hungarian Medical Research Council (ETT TUKEB IV/701-3/2022/EKU) and during all investigations, we followed the guidelines and regulations of the declaration of Helsinki. Healthy donors with informed consent were involved in our studies.

Note that full information on the approval of the study protocol must also be provided in the manuscript.

Field-specific reporting

Please select the one below that is the best fit for your research. If you are not sure, read the appropriate sections before making your selection.

☑ Life sciences ☐ Behavioural & social sciences ☐ Ecological, evolutionary & environmental sciences

For a reference copy of the document with all sections, see nature.com/documents/nr-reporting-summary-flat.pdf

Life sciences study design

All studies must disclose on these points even when the disclosure is negative.

Sample size	In the cell survival experiments, a minimum of three biological replicates, each with three technical replicates, were utilized. This sample size ensured that the error remained within 10% of the mean.
Data exclusions	No data were excluded.
Replication	Confirmed.
Randomization	Randomization in a cellular population is naturally given.
Blinding	Does not apply.

Behavioural & social sciences study design

All studies must disclose on these points even when the disclosure is negative.

Study description	
Research sample	
Sampling strategy	
Data collection	
Timing	
Data exclusions	
Non-participation	
Randomization	

Ecological, evolutionary & environmental sciences study design

All studies must disclose on these points even when the disclosure is negative.

Study description	
Research sample	
Sampling strategy	
Data collection	
Timing and spatial scale	
Data exclusions	
Reproducibility	
Randomization	
Blinding	

Did the study involve field work? ☐ Yes ☐ No

Field work, collection and transport

Field conditions	
Location	
Access & import/export	
Disturbance	

Reporting for specific materials, systems and methods

We require information from authors about some types of materials, experimental systems and methods used in many studies. Here, indicate whether each material, system or method listed is relevant to your study. If you are not sure if a list item applies to your research, read the appropriate section before selecting a response.

Materials & experimental systems

n/a	Involved in the study
☑	☐ Antibodies
☐	☑ Eukaryotic cell lines
☑	☐ Palaeontology and archaeology
☑	☐ Animals and other organisms
☑	☐ Clinical data
☑	☐ Dual use research of concern
☑	☐ Plants

Methods

n/a	Involved in the study
☑	☐ ChIP-seq
☑	☐ Flow cytometry
☑	☐ MRI-based neuroimaging

Antibodies

Antibodies used	
Validation	

Eukaryotic cell lines

Policy information about cell lines and Sex and Gender in Research

Cell line source(s)

MonoMac 6 human monocytic cells (DSMZ no.: ACC 124, Braunschweig, Germany) DSMZ cell lines bank (www.dsmz.de) which is a public cell line repository. The DSMZ is a non-profit research institute of the public Leibniz Association that is owned and subsidized by German federal and state governments.
Species: human (Homo sapiens)
Cell type: acute monocytic leukemia
Origin: established from the peripheral blood of a 64-year-old man with relapsed acute monocytic leukemia (AML FAB M5) in 1985 following myeloid metaplasia; carries t(9;11)(p22;q23) leading to KMT2A-MLLT3 (MLL-MLLT3; MLL-AF9) fusion gene; more information on this cell line on the following website: "www.monocyte.eu"; sister cell line of MONO-MAC-1 (DSM ACC 252); cells are used for the detection of pyrogens. Exome and RNA sequence data are available (see Ref 18187 and Exome sequence and RNA-Seq)
Biosafety level: 1
Morphology: single, round/multiformed cells or small clusters of cells in suspension, sometimes loosely adherent; 1-5% giant cells;

Authentication

This cell line was purchased from a public cell line repository, therefore only the cell morphology was tested by microscopy.

Mycoplasma contamination

no, checked by PCR test (QPCR MYCOPLASMA TEST KIT, 25 Tests, PanReac AppliChem)

Commonly misidentified lines
(See ICLAC register)

no commonly misidentified cell lines were used in this study

Palaeontology and Archaeology

Specimen provenance

Specimen deposition

Dating methods

☐ Tick this box to confirm that the raw and calibrated dates are available in the paper or in Supplementary Information.

Ethics oversight

Note that full information on the approval of the study protocol must also be provided in the manuscript.

Animals and other research organisms

Policy information about studies involving animals; ARRIVE guidelines recommended for reporting animal research, and Sex and Gender in Research

Laboratory animals

Wild animals

Reporting on sex

Field-collected samples

Ethics oversight

Note that full information on the approval of the study protocol must also be provided in the manuscript.

Clinical data

Policy information about clinical studies

All manuscripts should comply with the ICMJE guidelines for publication of clinical research and a completed CONSORT checklist must be included with all submissions.

Clinical trial registration

Study protocol

Data collection

Outcomes

Dual use research of concern

Policy information about dual use research of concern

Hazards

Could the accidental, deliberate or reckless misuse of agents or technologies generated in the work, or the application of information presented in the manuscript, pose a threat to:

No | Yes

- [ ] [ ] Public health
- [ ] [ ] National security
- [ ] [ ] Crops and/or livestock
- [ ] [ ] Ecosystems
- [ ] [ ] Any other significant area

Experiments of concern

Does the work involve any of these experiments of concern:

No | Yes

- [ ] [ ] Demonstrate how to render a vaccine ineffective
- [ ] [ ] Confer resistance to therapeutically useful antibiotics or antiviral agents
- [ ] [ ] Enhance the virulence of a pathogen or render a nonpathogen virulent
- [ ] [ ] Increase transmissibility of a pathogen
- [ ] [ ] Alter the host range of a pathogen
- [ ] [ ] Enable evasion of diagnostic/detection modalities
- [ ] [ ] Enable the weaponization of a biological agent or toxin
- [ ] [ ] Any other potentially harmful combination of experiments and agents

Plants

Seed stocks

Novel plant genotypes

Authentication

ChIP-seq

Data deposition

- [ ] Confirm that both raw and final processed data have been deposited in a public database such as GEO.
- [ ] Confirm that you have deposited or provided access to graph files (e.g. BED files) for the called peaks.

Data access links
May remain private before publication.

Files in database submission

Genome browser session
(e.g. UCSC)

Methodology

Replicates

Sequencing depth

Antibodies

Peak calling parameters

Data quality

Software

Flow Cytometry

Plots

Confirm that:

☐ The axis labels state the marker and fluorochrome used (e.g. CD4-FITC).

☐ The axis scales are clearly visible. Include numbers along axes only for bottom left plot of group (a 'group' is an analysis of identical markers).

☐ All plots are contour plots with outliers or pseudocolor plots.

☐ A numerical value for number of cells or percentage (with statistics) is provided.

Methodology

Sample preparation

Instrument

Software

Cell population abundance

Gating strategy

☐ Tick this box to confirm that a figure exemplifying the gating strategy is provided in the Supplementary Information.

Magnetic resonance imaging

Experimental design

Design type

Design specifications

Behavioral performance measures

Imaging type(s)

Field strength

Sequence & imaging parameters

Area of acquisition

Diffusion MRI ☐ Used ☐ Not used

Preprocessing

Preprocessing software

Normalization

Normalization template

Noise and artifact removal

Volume censoring

Statistical modeling & inference

Model type and settings

Effect(s) tested

Specify type of analysis: ☐ Whole brain ☐ ROI-based ☐ Both

Statistic type for inference

(See Eklund et al. 2016)

Correction

Models & analysis

n/a	Involved in the study
☐	☐ Functional and/or effective connectivity
☐	☐ Graph analysis
☐	☐ Multivariate modeling or predictive analysis

Functional and/or effective connectivity

Graph analysis

Multivariate modeling and predictive analysis

This checklist template is licensed under a Creative Commons Attribution 4.0 International License, which permits use, sharing, adaptation, distribution and reproduction in any medium or format, as long as you give appropriate credit to the original author(s) and the source, provide a link to the Creative Commons license, and indicate if changes were made. The images or other third party material in this article are included in the article's Creative Commons license, unless indicated otherwise in a credit line to the material. If material is not included in the article's Creative Commons license and your intended use is not permitted by statutory regulation or exceeds the permitted use, you will need to obtain permission directly from the copyright holder. To view a copy of this license, visit http://creativecommons.org/licenses/by/4.0/
